# Population Dynamics of *Ervilia castanea* (Montagu, 1803) Hints at Evolutionary Processes Shaping North‐East Atlantic Insular Sandy Habitats

**DOI:** 10.1002/ece3.71267

**Published:** 2025-06-03

**Authors:** Livia Sinigaglia, Lara Baptista, Manuel Curto, António Múrias Santos, Patrícia Madeira, Thapasya Vijayan, Harald Meimberg, Sérgio P. Ávila

**Affiliations:** ^1^ Institute of Integrative Nature Conservation Research, Department of Ecosystem Management, Climate and Biodiversity BOKU University Vienna Austria; ^2^ CIBIO, Centro de Investigação Em Biodiversidade e Recursos Genéticos InBIO Laboratório Associado, Pólo dos Açores Azores Portugal; ^3^ MPB‐Marine Palaeontology and Biogeography Lab University of the Azores Ponta Delgada Portugal; ^4^ Faculdade de Ciências da Universidade do Porto Porto Portugal; ^5^ Royal Netherlands Institute for Sea Research Texel the Netherlands; ^6^ Associação BIOPOLIS ‐ Rede de Investigação em Biodiversidade e Biologia Evolutiva Vairão Portugal; ^7^ CIBIO, Centro de Investigação em Biodiversidade e Recursos Genéticos, InBIO Laboratório Associado Universidade Do Porto Vairão Portugal; ^8^ UNESCO Chair – Land Within Sea: Biodiversity & Sustainability in Atlantic Islands Universidade dos Açores Ponta Delgada Portugal

**Keywords:** dispersal ability, *Ervilia castanea*, genetic population structure, oceanic islands, sandy habitats

## Abstract

Volcanic oceanic islands are some of the Earth's most geologically and ecologically dynamic habitats, where continuous volcanic activity and erosion lead to the formation of habitats that drastically change throughout their ontogeny. No more so than shallow‐water sandy habitats, which repetitively disappear and regenerate due to seasonal oceanographic and climatic eustatic sea‐level variations. For their inhabitants, these events translate into populations being cyclically removed or experiencing drastic reductions in population size, where the outcome often depends on the specific life‐history modes of the species, determining their dispersal and colonization potential and, ultimately, their survival ability. Therefore, population genetic patterns of marine shallow‐water infaunal species can provide powerful clues to such outcomes, as well as how specific geological and ecological settings determine the genetic structure of the species. We herewith test the population structure of the marine infaunal bivalve *Ervilia castanea* (Montagu, 1803) in the sandy habitats of the Azores and Madeira Archipelagos (Northeast and Central Atlantic Ocean), by comparing insular populations with conspecifics from the nearest continental shores in mainland Europe. Little to no genetic structure was observed between insular populations with both nuclear microsatellites and the mitochondrial cytochrome *c* oxidase subunit I. Moreover, deviations in the Hardy–Weinberg Equilibrium of insular populations suggest the existence of archipelago‐specific processes. The high dispersal ability of *E. castanea* combined with the ephemeral nature of oceanic shallow‐water sandy habitats likely made each population composed of individuals from multiple sources. The high prevalence of null alleles and gene duplication hint at the potential occurrence of recent polyploidization events that require further investigation. Moreover, we found evidence of hyperdiversity among the markers used which may constrain the detection of more detailed patterns. We herewith demonstrate the uniqueness of insular environmental settings and inquire further into the evolutionary and biogeographic patterns of marine shallow‐water infaunal species from volcanic oceanic islands.

## Graphical abstract

Shallow‐water sandy habitats of volcanic oceanic islands are among the most geologically and ecologically dynamic marine habitats on Earth. We investigate the population genetic patterns of *Ervilia castanea* , an infaunal marine bivalve with a planktotrophic larvae inhabiting Macaronesian shallow‐water sandy habitats. Little to no genetic structure was observed between insular populations alongside a unique genetic signature that inquires further investigation.

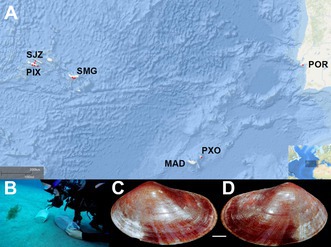

## 1 Introduction

Volcanic oceanic islands are exposed to destructive ocean forces from the moment they breach the sea surface until they eventually disappear due to marine erosion and subsidence. Within this time frame, constructional and destructive processes happen side by side, rendering these habitats excellent natural laboratories to observe ongoing geobiological processes (Ramalho et al. 2013). Geologically short‐lived compared to their continental counterparts, these confined environments belong to a system of large‐scale geological and oceanographic features, such as undersea ridges, seamount chains, boundaries, and convergence zones, that are biogeographically connected and serve as critical stepping stones for species dispersal across the ocean (Anderson et al. 2013). The distinctive nature of oceanic volcanic islands shapes unique biological assemblages over time, often characterized by high proportions of species broadly distributed with great dispersal abilities (Anderson et al. 2013), alongside many endemic, non‐planktotrophic species, mostly with low dispersal capacities (Ávila 2000a, 2006).

On a local scale, the interaction between physical and habitat‐related factors such as bathymetry and topography, wave disturbance, bed shear stress, and nutrient supply from oceanic currents, can be a strong predictor of species abundance, biodiversity, and the overall community structure and function (Anderson et al. 2013). The soft‐sediment habitats of oceanic islands originate from a combination of volcanoclastic and biogenic materials shaped by subaerial erosion and high wave energy, particularly from large oceanic swells (Ramalho et al. 2013). Consequently, oceanic islands' seabed forms are extremely dynamic around the shelf (Mitchell et al. 2022; Zhao et al. 2022) and support impoverished assemblages with reduced trophic structures (Anderson et al. 2013). Seasonally, storms and down‐welling currents cause sediment depletion in nearshore areas, transporting sediments to the middle and outer shelf (Ávila, Madeira, Da Silva, et al. 2008; Zhao et al. 2022). On a geological timescale, in contrast to continental shelves, which can accumulate a significant amount of sediment, shelves from reefless oceanic islands may go through periods of complete sediment starvation (Anderson et al. 2013). In the last 2.5 million years (Ma) (Quaternary), major climate fluctuations between glacial and interglacial conditions have driven significant eustatic changes in sea level with amplitudes as high as 130 m (Miller et al. 2011). In addition, many islands lack major riverine systems, and for most volcanic oceanic islands, shelf sediments are lost to greater depths whenever sea level drops below the insular shelf edge during glacial periods (Ávila, Madeira, Da Silva, et al. 2008; Ávila, Melo, Silva, et al. 2015).

This dynamic has a crucial impact on marine shallow‐water infaunal species which may go through cycles of repeated local disappearance/extirpation of populations or even the extinction of single island marine endemics (Ávila, Madeira, Da Silva, et al. 2008; Ávila et al. 2018). Cases of local disappearance of shallow endobenthic species from oceanic islands have been documented since the last glacial episode (Ávila, Madeira, Da Silva, et al. 2008; Ávila et al. 2020). Hard substrate‐associated species can survive glacial episodes, being able to cope with sea level drops and steep island gradients (Ávila, Madeira, Da Silva, et al. 2008; Ávila, Madeira, Mendes, et al. 2008; Ávila, Madeira, et al. 2009; Ávila, Melo, Silva, et al. 2015; Ávila 2013). Such cyclical extirpation events explain the low endemism levels of marine littoral taxa associated with soft sediments (Ávila, Melo, Silva, et al. 2015; Ávila et al. 2018, 2020) compared to hard substrate species in oceanic volcanic systems. In general, depending on the insular littoral area available (*sensu* Ávila et al. 2018, 2019), sandy beaches of oceanic islands may pass from being notoriously devoid of life during glacial periods to being promoters of habitat diversity, increased species carrying capacity, and higher speciation rates during interglacial periods (Ávila et al. 2019). Opportunistic life strategies, including high fecundity, fast growth rates, and long‐distance dispersal ability, are positively selected, enabling the quick recolonization of sediment patches (Anderson et al. 2013). Species with planktotrophic larvae, which usually have larger geographical and ecological ranges and higher genetic diversity, are less susceptible to extinction and potentially occupy life‐depleted sediments (Scheltema and Williams 1983). The interaction between intermittent dispersal and colonization may promote both the origination and extinction of new lineages. As high levels of gene flow are sustained, most marine species with high dispersal abilities maintain highly connected populations even between isolated archipelagos (Pinheiro et al. 2017).

**FIGURE 1:**
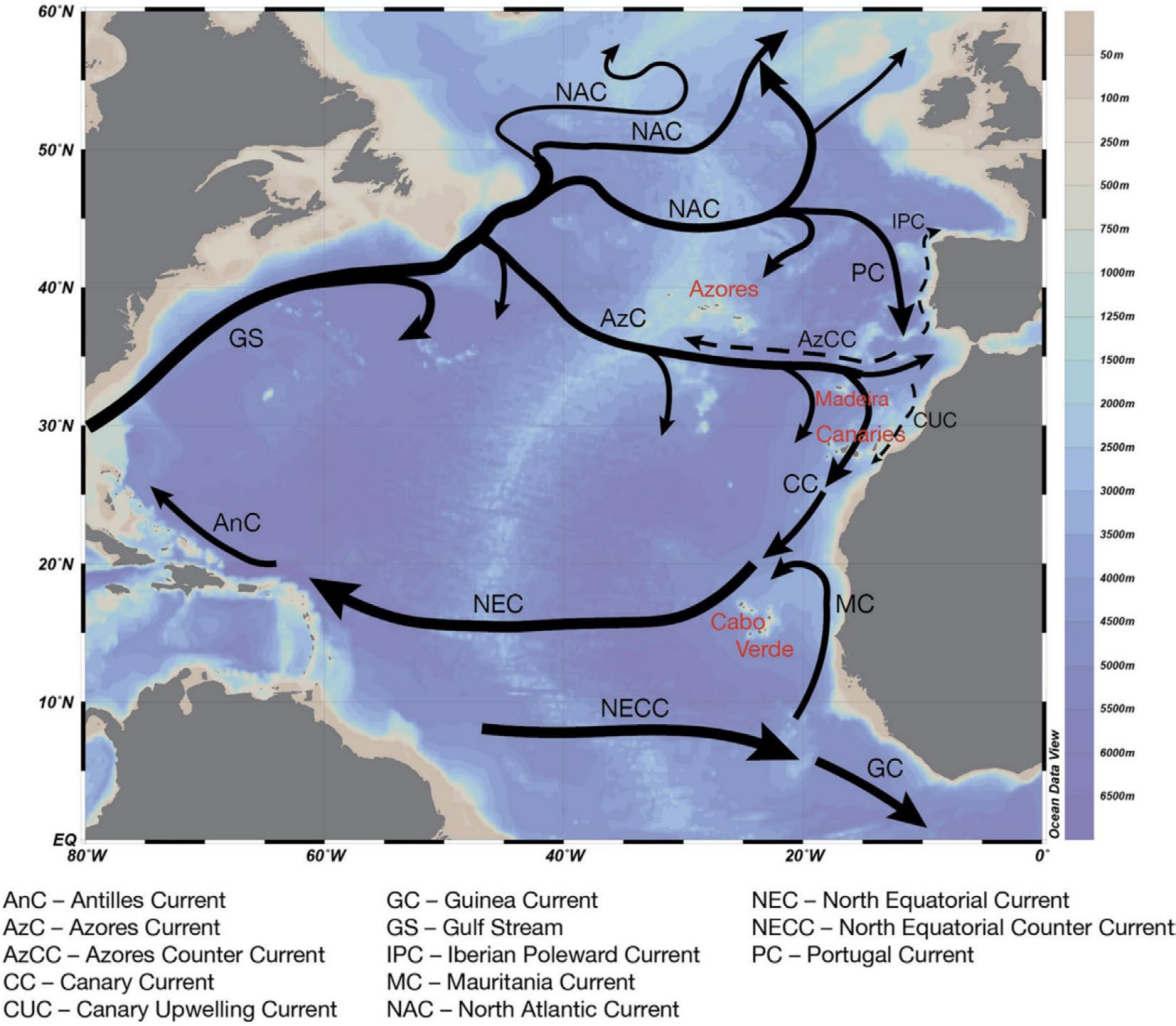
Main surface circulation patterns in the North and Central Atlantic Ocean. All Macaronesian Archipelagos but Selvagens are identified: Azores, Madeira, Canaries and Cabo Verde. Individuals from mainland Portugal were also included in this study. Gulf Stream recirculation and circulation north of 40° N based on Daniault et al. (2016). Dashed lines represent seasonal currents and are exemplified by the Iberian Poleward Current and Canary Upwelling Current. The Azores Counter Current (AzCC) is dominantly a subsurface flow and therefore also shown as dashed line; adapted from Baptista et al. (2021).

The NE Atlantic archipelagos of the Azores and Madeira might offer some insight on the role of marine organisms with planktotrophic larvae in the colonization of sandy habitats in oceanic islands. These archipelagos belong to the biogeographical subtropical Mediterranean–Moroccan Province (*sensu* Ávila et al. 2016) and have been further sub‐grouped into the Azores Ecoregion and the Webbnesia Ecoregion, the latter including the Madeira, Selvagens, and Canaries archipelagos (Freitas et al. 2019). Never having been connected to continental landmasses, these archipelagos are assumed to have been colonized by long‐distance oceanic dispersal processes and crucially shaped by past sea‐surface currents and circulation patterns (Jokiel 1990). Despite the present system of sea‐surface currents in the North Atlantic predominantly flowing in an eastward direction in the Azores region, most of the fauna originates from the opposite direction (Figure 1).

The Azorean islands rise from a 2000 m bathymetric anomaly known as the “Azores Plateau” (Needham and Francheteau 1974), where the North American, Eurasian, and Nubian plates meet (Ramalho et al. 2017). The Azores are the most remote archipelago in the North Atlantic Ocean, located 1370 km from the Iberian Peninsula. These wide distances are determinant for its isolation and consequent impoverished biological assemblages (Freitas et al. 2019). Compared to other NE Atlantic Archipelagos, most islands in the Azores (eight out of nine) have been classified as geologically ‘immature’ or ‘young’ (Ávila et al. 2019). The Madeiran Archipelago consists of two principal islands (Madeira and Porto Santo), which are instead geologically ‘old’ or ‘mature’ (Ávila et al. 2019). These islands correspond to the south‐western termination of a broad alignment of scattered seamounts and volcanic ridges, 900 km from the Iberian Peninsula (Geldmacher et al. 2000). Located on a 140 Ma old oceanic crust, this archipelago rises from more than 4000 m deep waters, reaching an altitude of 1862 m above sea level (Geldmacher et al. 2000). While Madeira Island lacks wide sandy beaches, these are alternatively found in the smaller eastern volcanic island of Porto Santo (69 km^2^).

*Ervilia castanea* (Montagu, 1803) is an endobenthic suspension feeder bivalve belonging to the family Semelidae (Superfamily Tellinoidea), characterized by the development of cruciform muscles and very long siphons that allow burrowing deep into the sediment (Mikkelsen 2011). *E. castanea* is a very successful colonizer of mobile sandy substrates and is mostly found inhabiting the first centimeter of well‐sorted sediments overlain by clean, nutrient‐poor, oceanic waters (Morton *et aI*., 1998) reaching depths of 100 m (Ávila, unpublished data). Individuals of this gonochoric species have a high potential reproductive output, with maturation occurring at a length of 3.5 mm for females and 5.5 mm for males (Morton 1990). Although no study to date has investigated the specific ontogenetic development of *E. castanea* , semelids are known to produce planktonic veliger larvae (Mikkelsen and Bieler 2008).

This bivalve is currently known to range from ‘open’ ocean areas south of the British Isles to Atlantic coasts of Portugal, the Mediterranean Sea (Babio and Bonnin 1987; Macedo et al. 1999; Morton 1990), and the Atlantic Archipelagos of the Azores, Madeira (MolluscaBase eds. 2024), Selvagens and Canaries (Ávila, unpublished data). According to Cosel & Gofas (2019), this species has locally disappeared from West Africa, with Holocene empty shells and valves being reported from Morocco, West Sahara, Mauritania and Senegal, in water depths of 40–200 m. Fossils of this taxa are also reported from the Pleistocene (Last Interglacial) of Santa Maria Island, Azores (119–126 kyr) (Ávila et al. 2002; Ávila, Rebelo, et al. 2010), from tsunami deposits of the glacial MIS 6 in Canaries Archipelago (170–120 kyr) (Coello Bravo et al. 2014), and the MIS5e deposits of Tenerife Island (Canaries) (Martín‐González et al. 2016), showing long‐time persistence in these areas. Today, this species is the most common bivalve in the Azorean shallow sandy bottoms (Morton 1990), reaching densities of ~2000 individuals/kg of sand and occupying depths from 5 to 20 m (Ávila, Guedes, et al. 2010).

Both Madeira and Azores archipelagos display biologically impoverished beaches, probably consequent to the sea level dropping below the 80 m isobath 12 times during the last million years (Ávila 2013). Such events caused cyclical extirpations of most, if not all, of the soft substrate‐associated littoral marine species (Ávila et al. 2019) or severe “bottleneck” effects that resulted in accentuated decreases in population size (Ávila et al. 2020). Consequently, Ávila et al. (2019, 2020) predicted lower genetic diversity of the insular populations of shallow‐water species associated with sandy bottoms when compared with continental conspecific populations.

We hereby propose a molecular approach to using the infaunal sandy bivalve *Ervilia castanea* as a model, aiming to: (i) characterize the molecular diversity among insular and continental populations; (ii) explore the effects of a planktotrophic larval dispersal mode on population dynamics in sandy habitats in both islands and the mainland. To answer such questions, we investigate the population genetics inferred from both nuclear microsatellites and the mitochondrial cytochrome *c* oxidase subunit I (COI) gene.

## 2 Materials and Methods

### 2.1 Sample Collection

**FIGURE 2:**
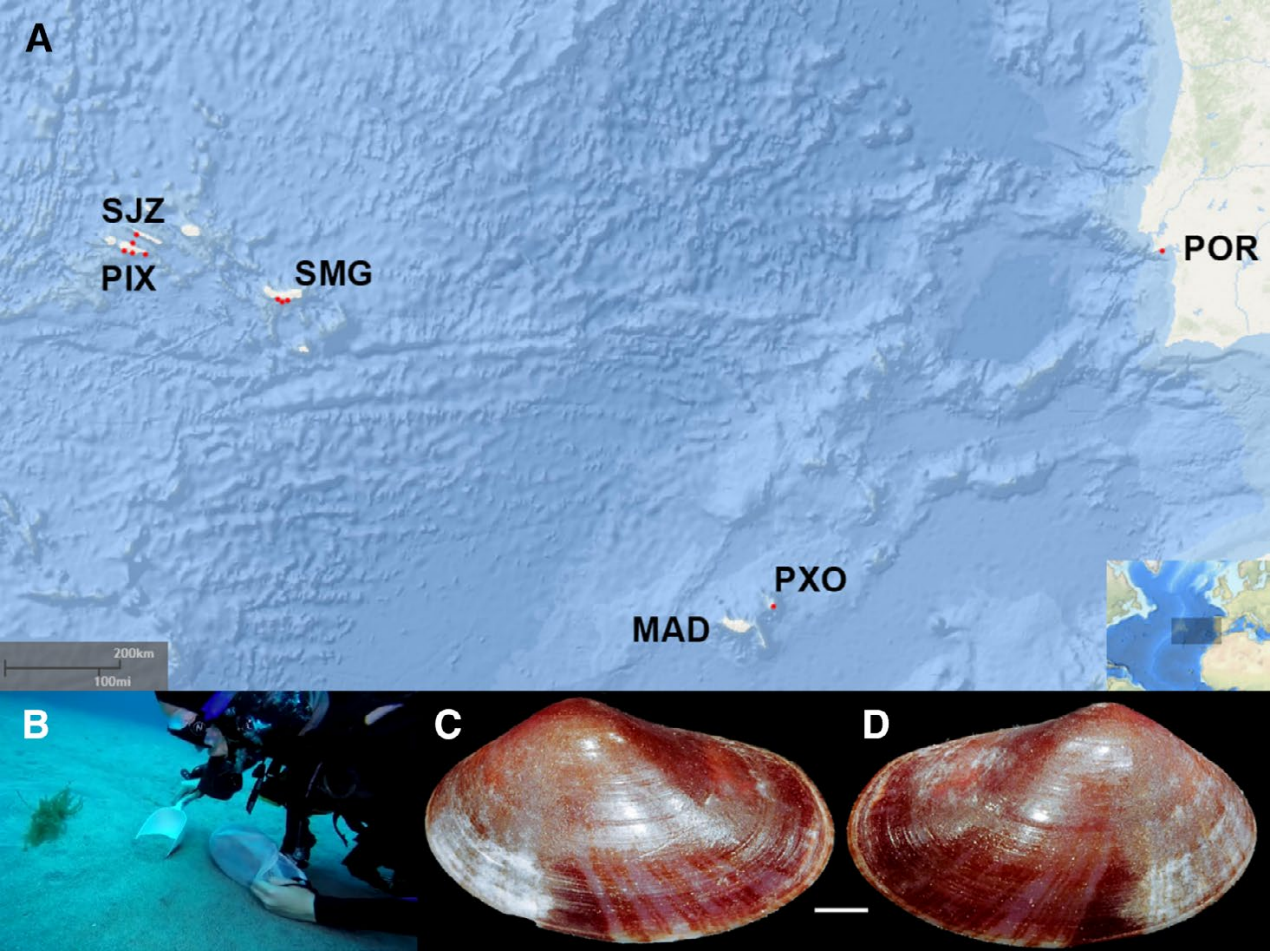
(A) Study area and sampling locations in the NE Atlantic Ocean (red dots). Geographical location of the Azores Archipelago (Portugal) and the sampled islands of São Jorge (SJZ), Pico (PIX) and São Miguel (SMG); Madeira Archipelago (MAD) with two sampled locations in Porto Santo Island (PXO); mainland Portugal (POR) with sampling location in Sesimbra. General Bathymetric Chart of the Oceans (GEBCO) Map derived from NOAA/NCEI Bathymetric Data Viewer by NOAA/NCEI https://www.ncei.noaa.gov/maps/bathymetry/. (B) Sampling methodology through SCUBA diving. (C, D) *Ervilia castanea* (Montagu, 1803) from DBUA 157, collected off Cais do Tagarete, Vila Franca do Campo, São Miguel Island, Azores at 52 m depth. (C) Left valve, external view. (D) Right valve, external view. Scale: 1 mm.

A total of 308 individuals of *Ervilia castanea* from different locations in the Azores, Madeira, and mainland Portugal were used in this study (Figure 2; Table 1). Fresh samples were directly collected by SCUBA diving at depths ranging from 8 to 20 m, whereby the bivalve was found burrowing in the first centimeter of sand and stored in absolute ethanol. Permits for sampling were issued by the respective authorities in the Azores (Região Autónoma dos Açores, Secretaria Regional do Mar e das Pescas, Direção Regional dos Assuntos do Mar, AMP/2021/009, CCIP 29/2021/DRCT), Madeira (Licença n. 2/2022 PS; 3/2022 M), and Sesimbra (ICNF—Licença S‐023454/2023).

**TABLE 1:** *Ervilia castanea* individuals sampled and successfully processed in the laboratory.

Individuals	Location	Coordinates (latitude; longitude)
41	Calheta, PIX, AZO	38.40254; −28.068351
41	Fonte, PIX, AZO	38.41236; −28.2827
11	Furna, PIX, AZO	38.538574; −28.337769
45	São Caetano, PIX, AZO	38.425649; −28.421754
11	Morro da Enseada, SJZ, AZO	38.699163; −28.228292
34	Dori, SMG, AZO	37.746271; −25.623826
33	Ilhéu de Vila Franca, SMG, AZO	37.705343; −25.440728
13	Lagoa, SMG, AZO	37.740769; −25.572124
49	Ilhéu de Cima, PXO, MAD	33.053864; −16.284299
5	Porto, PXO, MAD	33.061813; −16.308117
10	Sesimbra, POR	38.433717; −9.116535

*Note:* Geographical coordinates of the sampling locations in decimal degrees are reported.

Abbreviations: AZO, Azores; AZO, Azores; MAD, Madeira; PIX, Pico; POR, Portugal (mainland); PXO, Porto Santo; SJZ, São Jorge; SMG, São Miguel.

### 2.2 DNA Isolation Protocol

Total genomic DNA (gDNA) was extracted from the entire animal after being removed from the shell, and the excess preservation ethanol was dried with a paper towel. The extraction protocol follows guidelines from Sinigaglia et al. (2024) and details can be found therein. An electrophoretic run in agarose gel 1.5% at 80 V for 30 min was performed to evaluate DNA integrity. Samples with clear bands of *circa* 23,000 bp were considered good quality extracts and used in the further molecular procedures.

### 2.3 GBAS Analysis

#### 2.3.1 Library Preparation—Illumina Sequencing

Microsatellite markers were genotyped using the SSR‐GBAS protocol (Curto et al. 2019) allowing the recovery of high levels of polymorphism while implementing an automated and reproducible allele‐calling approach. This approach removes biases that might prevent the concatenation of datasets from independent analyses (Tibihika et al. 2019). Markers were developed *de novo* following Sinigaglia et al. (2024). The newly designed primer pairs that successfully generated amplicons under the expected size range (in between 300 and 500 bp) were combined in two primer mixes containing 12 markers, each with a final concentration of 1 μM (Table S1, Additional Files).

#### 2.3.2 Multiplex PCR and Sequencing

Multiplex PCR amplification and indexing conditions follow Sinigaglia et al. (2024). The resulting indexed‐PCR products were then sent to Biozentrum der LMU München, Lehrstuhl Genetik (München, Germany) for Illumina MiSeq run for PE 300 bp sequencing or to Novogene UK Company (Cambridge, United Kingdom) for NovaSeq 6000 run for PE 300 bp sequencing.

#### 2.3.3 SSR Data Analysis

Raw FASTQ sequence data (Genbank BioProject PRJNA1137574) underwent quality control and merging procedures with FastQC v0.11.9 (Andrews 2010), Trimmomatic v0.39 (Bolger et al. 2014), Usearch v11 (Untergasser et al. 2012). Genotyping and analysis of each sample and SSR locus were conducted with the SSR_GBS_pipeline scripts available at GitHub (https://github.com/mcurto/SSR‐GBS‐pipeline). Marker plots generated during the allele length calling step allowed for manual verification of marker duplication or other potential errors. Allele calls were based on whole amplicon information (WAI) that uses sequence identity integrating both SNP and length variation. The resulting codominant matrix was used for further population genetic analyses.

#### 2.3.4 Quantifying Genetic Diversity and Genetic Structure From SSR Data

The codominant matrix was imported and manually checked in Excel, and samples and markers containing more than 60% missing data were removed from the analysis. GenAlEx v6.5 (Peakall and Smouse 2006) allowed estimates of deviations from Hardy–Weinberg Equilibrium (HWE) in the WAI dataset, the number of alleles (*N*_a_) and effective alleles (*N*_e_) information index (I), observed heterozygosity (*H*_o_) and expected heterozygosity (*H*_e_) heterozygosity, and population inbreeding coefficient (*F*).

FreeNa (Chapuis and Estoup 2007) was used to estimate the null allele frequencies per population, considering 100 bootstraps. To comply with the software requirements of a maximum of 98 alleles per locus, the length SSR matrix was used instead of the WAI dataset, which combines length and SNPs with over 100 alleles per locus.

A hierarchical analysis of molecular variance (AMOVA) was performed in GenAlEx to evaluate the differentiation between populations and regions (Azores, Madeira and Sesimbra). Pairwise F_ST_ genetic distance was calculated to assess evolutionary divergence between the populations. Genetic distance patterns between individuals were evaluated in a principal coordinate analysis (PCoA). To analyze the retrieved allelic pattern, the number of alleles and the number of alleles with frequency above 5% were calculated per marker, while the number of private alleles was calculated per population using the GenAlEx frequency function. A discriminant analysis of principal components (DAPC) was conducted using the adegenet package (Jombart et al. 2018) in R to investigate the genetic differentiation among populations. To optimize the balance between minimizing overfitting and capturing meaningful genetic structure, we employed the optim.a.score function, which selects the optimal number of principal component axes to retain based on cross‐validation. This approach ensures the selection of the optimum number of axes that maximizes the discriminatory power while avoiding the inclusion of noise. We performed an initial analysis with all populations that retained 70 axes. To evaluate further patterns within the archipelagos, an additional analysis without the mainland population Sesimbra was done, and 45 axes were retained.

The software STRUCTURE v2.3.4 (Pritchard et al. 2000) was used to produce a probability of assignment of each individual to a hypothetical group with assumptions of HWE. With the number of clusters (K) varying between 1 and 11, STRUCTURE ran for 10 independent replicates for 100,000 generations, following a burn‐in period of 100,000 (default settings were maintained for the admixture model and correlated allele frequencies). The online program Structure Harvester (Earl and Von Holdt 2012) was used to validate multiple *K*‐values for optimal detection of genetic structure, according to the Delta‐K method and inferring the K that best suits the data from hundreds of iterations. The results from STRUCTURE across the K‐values were summarized and graphically displayed, resorting to the online pipeline CLUMPAK (Kopelman et al. 2015).

BayesAss v 3.0 (Rannala 2007) was used to estimate the migration rates between the sampled populations. The system ran for 10,000,000 iterations with a 1,000,000 burn‐in. The mixing parameters for allele frequencies and inbreeding coefficient were adjusted respectively to 0.4 and 0.5 so that an acceptance rate between 20% and 60% could be reached. The parameters were then checked in Tracer v 1.7.1 (Rambaut et al. 2018) for ESS values higher than 100.

BOTTLENECK v 1.2.02 (Piry et al. 1999) was used to test whether any of the studied populations experienced a significant reduction in observed heterozygosity compared to the expectation under the mutation‐drift equilibrium indicating the existence of a bottleneck. Expected heterozygosity values were estimated using the infinite allele model, and deviations were tested using the one‐tailed Wilcoxon test.

### 2.4 Mitochondrial Analysis

#### 2.4.1 *Molecular Marker Amplification–*Sequencing of mtDNA (COI)

COI was amplified in 20 μL when samples were sent to the commercial facility AGENTA GeneWiz (Leipzig, Germany) or 10 μL when samples were sequenced at the Centre for Molecular Analyses (CTM from CIBIO‐InBIO Research Centre, Vairão, Portugal). The amplification of COI was achieved with the primers jgLCO1490/jgHCO2198 (Geller et al. 2013). Details about the PCR reactions, cycling conditions, and Sanger sequencing of the COI dataset can be found in Supporting Information.

#### 2.4.2 mtDNA COI Analysis

COI chromatograms were manually checked for the presence of misreads with Geneious Prime 2022.2.2. The revised sequences were then inspected with AliView (Larsson 2014) for the existence of stop codons. All the sequences generated in this study were deposited in GenBank under the accession codes PP976992‐PP977008. The COI dataset was aligned with Geneious Prime v2022.2.2 using global alignment with free end gaps and a cost matrix of 65% similarity. Sequences under 300 bp were excluded from the study, and the remaining were trimmed to 406 bp to prevent the inferring of any evolutionary divergence merely due to differences in sequence length. A haplotype network was inferred with a statistical parsimony haplotype network at the 95% connection limit (TCS; Clement et al. 2000). The output was rendered using the web‐based program tcsBU (dos Santos et al. 2016), which allowed the depiction of the geographic locations along with the genetic structure retrieved by TCS.

## 3 Results

### 3.1 Population Genetic Structure (SSR) Analyses

For the SSR marker discovery, MiSeq runs for two *E. castanea* samples (SP2 and EV1) produced 11,369,406 and 4,620,456 raw reads respectively. After the trimming and merging steps, 5,061,638 reads resulted for SP2 and 2,122,895 for EV1. Reads containing SSR repeats were used to design 50 primer pairs. Twenty‐four primer pairs were successful, while 26 failed to amplify in the single PCR test. The successful 24 primer pairs were included in the two multiplex primer mixes (12 primer pairs each, Table S1, Additional Files).

#### 3.1.1 Genetic Diversity Measures

In total, 308 samples successfully passed the PCR step and were sequenced with Illumina and NovaSeq technologies. The Illumina runs successfully produced 19,290,180 and 6,118,697 paired raw reads for Miseq and NovaSeq, respectively. After the quality control steps and filtering for missing data, a final matrix of 12 SSR loci and 266 individuals was obtained (Table S2, Additional Files). Markers showed a surprisingly high number of alleles ranging from 95 to 262 (Table 2). Interestingly, just a few of these (0–6 per locus) had a frequency above 5%, indicating that this variation was not shared across individuals (Table 2). This was reflected in the large number of private alleles per population that ranged between 59% and 100% of the total number of alleles (Table 2).

**TABLE 2:** Allele frequency statistics.

Population	No. alleles	No. private alleles
Calheta, PIX, AZO	12.33	8.33
Fonte, PIX, AZO	30.17	21.08
Furna, PIX, AZO	10.25	6.5
Ilhéu de Cima, PXO, MAD	34.41	27.83
São Caetano, PIX, AZO	34.08	26.67
Morro da Enseada, SJZ, AZO	10.25	6.08
Dori, SMG, AZO	27.08	19.92
Ilhéu V. Franca, SMG, AZO	23.67	17.67
Lagoa, SMG, AZO	11.33	7.17
Porto, PXO, MAD	4.17	2.92
Sesimbra, POR	3.42	3.42

Locus	Nr. alleles	Freq ≥ 5%
SP2EC6_ATTGT	122	6
Ev1_TATGA	208	2
32SP2EC_TATGA	262	1
SP2EC9_ATATC	190	2
23SP2EC_ATGA	101	3
29SP2EC_TGTT	131	2
SP2EC57_3mer	160	4
48SP2EC_ATTGA	148	1
SP2EC7_TCAAG	143	2
Ev2_TTGAA	95	0
SP2EC8_ATATG	129	0
Ev8_AATTG	161	0

*Note:* Mean number of total and private alleles analyzed per population. Number of alleles and number of different alleles with frequencies higher than 5% (≥ 5%) are analyzed per marker. For populations' abbreviations, please see the legend of Table 1.

Analysis of deviations from HWE showed significant values for most loci in the Azorean populations, while they were mostly non‐significant for Sesimbra populations (Table 3). The population of Porto (Porto Santo) was excluded from this analysis as only five individuals represented the population and were thus considered to potentially bias the result when compared to the remaining populations.

**TABLE 3:** Hardy–Weinberg equilibrium (HWE) tested for all analyzed loci and populations of *Ervilia castanea* .

Population	SP2EC6_ATTGT	Ev1_TATGA	32SP2EC_TATGA	SP2EC9_ATATC	23SP2EC_ATGA	29SP2EC_TGTT	SP2EC57_3mer	48SP2EC_ATTGA	SP2EC7_TCAAG	Ev2_TTGAA	SP2EC8_ATATG	Ev8_AATTG
Calheta, PIX, AZO	*	*	*	*	ns	*	*	*	*	*	*	*
Fonte, PIX, AZO	*	*	*	*	*	*	*	*	*	*	*	*
Furna, PIX, AZO	ns	ns	ns	*	*	*	*	*	*	ns	*	*
São Caetano, PIX, AZO	*	*	*	*	*	*	*	*	*	*	*	*
Morro da Enseada, SJZ, AZO	ns	*	*	*	ns	*	*	*	ns	*	*	*
Dori, SMG, AZO	*	*	*	*	*	*	*	*	*	*	*	*
Ilhéu V. Franca, SMG, AZO	*	*	*	*	*	*	*	*	*	*	*	*
Lagoa, SMG, AZO	ns	*	*	*	ns	*	*	*	*	*	*	*
Ilhéu de Cima, PXO, MAD	*	*	*	*	*	*	*	*	*	*	*	*
Sesimbra, POR	ns	Mono	Mono	*	ns	*	*	ns	ns	Mono	Mono	*

*Note:* ‘*’ Indicates significance, ‘ns’ indicates non‐significance, ‘Mono’ indicates monomorphic marker. For populations' abbreviations, please see legend of Table 1.

Observed heterozygosity (*H*_o_) values were lower than expected ones (u*H*_e_) for all populations (Table 4) with positive F values in all locations, which may indicate the presence of null alleles. This was confirmed by a relatively high prevalence of null alleles, ranging between 0% to 45% and a mean of 18% of null alleles per locus and population (Table S3). Pairwise F_ST_ genetic distance measures showed the greatest distance between Sesimbra (mainland Portugal) and the NE Atlantic archipelagos (mean 0.3, Table 5).

**TABLE 4:** Mean and standard error (SE) over loci for each population of *Ervilia castanea* : Sample size (*N*), No. alleles (*N*_a_), No. effective alleles (*N*_e_), information index (I), observed heterozygosity (*H*_o_) and unbiased expected heterozygosity (u*H*_e_), and fixation index (F).

*Population*	*Island*	*Archipelago*		*N*	*Na*	*Ne*	*I*	*Ho*	*uHe*	*F*
Calheta	Pico	Azores	Mean	11.000	13.385	10.635	2.353	0.385	0.908	0.560
			SE	0.641	1.563	1.641	0.148	0.062	0.031	0.067
Fonte	Pico	Azores	Mean	32.385	33.692	22.238	3.109	0.397	0.924	0.560
			SE	1.917	4.816	4.846	0.203	0.062	0.029	0.065
Furna	Pico	Azores	Mean	9.308	10.846	9.083	2.173	0.349	0.890	0.587
			SE	0.286	1.114	1.179	0.148	0.062	0.038	0.065
São Caetano	Pico	Azores	Mean	35.538	37.769	22.864	3.185	0.404	0.921	0.550
			SE	1.828	5.177	5.300	0.197	0.067	0.028	0.072
Morro da Enseada	São Jorge	Azores	Mean	9.385	11.154	8.925	2.194	0.410	0.905	0.526
			SE	0.525	1.260	1.381	0.128	0.076	0.023	0.083
Dori	São Miguel	Azores	Mean	28.385	29.846	18.124	2.988	0.420	0.918	0.532
			SE	1.375	3.889	4.048	0.176	0.067	0.027	0.071
Ilhéu V. Franca	São Miguel	Azores	Mean	27.615	26.615	15.840	2.758	0.367	0.867	0.558
			SE	1.323	3.758	4.216	0.225	0.061	0.049	0.065
Lagoa	São Miguel	Azores	Mean	10.615	12.385	9.766	2.250	0.424	0.880	0.489
			SE	0.460	1.338	1.575	0.161	0.073	0.045	0.090
Ilhéu de Cima	Porto Santo	Madeira	Mean	36.692	38.615	24.956	3.272	0.320	0.944	0.660
			SE	2.781	5.623	5.653	0.175	0.062	0.015	0.063
Porto	Porto Santo	Madeira	Mean	3.308	4.615	4.277	1.356	0.349	0.792	0.520
			SE	0.365	0.712	0.698	0.169	0.094	0.073	0.109
Sesimbra	Portugal	Portugal	Mean	5.538	3.462	2.554	0.930	0.208	0.504	0.551
			SE	1.191	0.829	0.631	0.205	0.069	0.102	0.104

*Note:* For populations' abbreviations, please see the legend of Table 1.

**TABLE 5:** Pairwise *F*_ST_ genetic distance between the sampled *Ervilia castanea* populations. The different shades of red are relative to the resulting range of values, with the darkest colouring indicating the highest values.

Calheta, PIX, AZO	Fonte, PIX, AZO	Furna, PIX, AZO	São Caetano, PIX, AZO	Morro da Enseada, SJZ, AZO	Dori, SMG, AZO	Ilhéu V. Franca, SMG, AZO	Lagoa, SMG, AZO	Ilhéu de Cima, PXO, MAD	
0.000									Calheta, PIX, AZO
0.026	0.000								Fonte, PIX, AZO
0.043	0.029	0.000							Furna, PIX, AZO
0.025	0.013	0.030	0.000						São Caetano, PIX, AZO
0.047	0.031	0.052	0.034	0.000					Morro da Enseada, SJZ, AZO
0.028	0.018	0.030	0.013	0.037	0.000				Dori, SMG, AZO
0.031	0.019	0.032	0.017	0.039	0.020	0.000			Ilhéu V. Franca, SMG, AZO
0.039	0.029	0.042	0.026	0.048	0.030	0.028	0.000		Lagoa, SMG, AZO
0.039	0.024	0.042	0.025	0.039	0.028	0.035	0.044	0.000	Ilhéu de Cima, PXO, MAD
0.277	0.261	0.286	0.262	0.279	0.264	0.287	0.290	0.252	Sesimbra, POR

*Note:* Heat map shows darker red cells when a relatively higher distance value is found. For abbreviations, please see legend of Table 1.

#### 3.1.2 Genetic and Spatial Structure Analyses

The AMOVA analysis, performed among regions (Azores, Madeira, and mainland Portugal) and populations, showed that only 6% of the variation detected can be explained between regions, while 63% of it lies among individuals (Table 6).

**TABLE 6:** Hierarchical analysis of molecular variance (AMOVA) among *Ervilia castanea* .

Source	df	SS	MS	Est. Var	%
Among regions	2	88.157	44.079	0.360	6
Among populations	7	67.589	9.656	0.019	0
Among individuals	251	2188.544	8.719	3.519	63
Within individuals	261	439.000	1.682	1.682	30
Total	521	2783.291		5.580	100

*Note:* Analysis performed among populations and geographical regions (Azores, Madeira, mainland Portugal), with 999 permutations.

**FIGURE 3:**
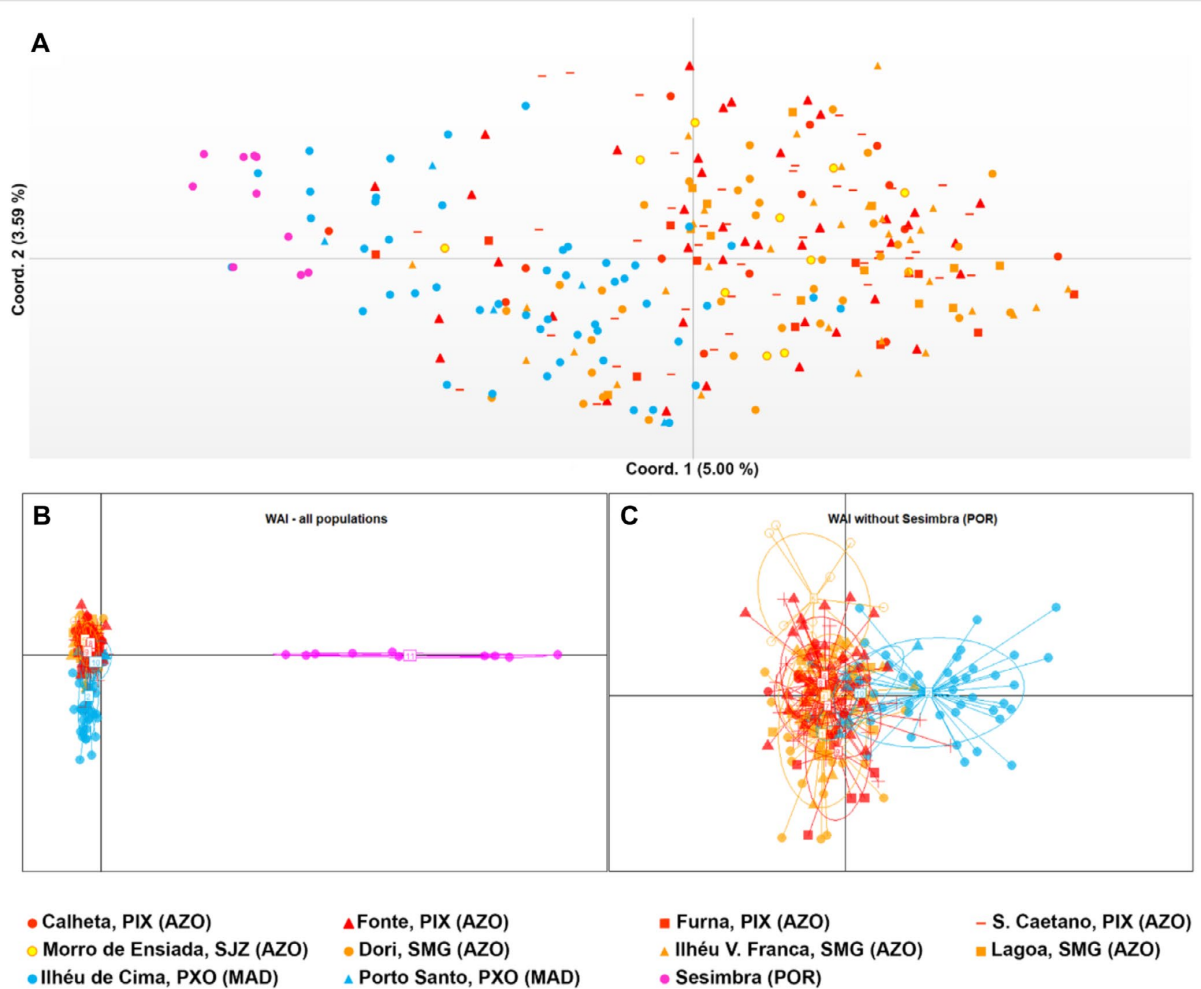
(A) Principal coordinates analysis from complete WAI dataset of *Ervilia castanea* from the sampled populations in the Azores (AZO), Madeira (MAD), and mainland Portugal (POR). Variance explained by axis 1 (5.00%) and axis 2 (3.59%). PCoA conducted as implemented in GenAlEx v6.5; populations in study are coded by color (A). (B, C) Discriminant analysis of principal components (DAPC) from WAI dataset of *Ervilia castanea* from the sampled populations in the Azores (AZO), Madeira (MAD), and mainland Portugal (POR). Analyses were conducted for all populations (B) and without the population of Sesimbra (mainland) (C). The optimal number of PCoA axes explaining the variation was 60. For populations' abbreviations, please see legend of Table 1.

The PCoA revealed no clear distinction between Azorean, Madeiran, and Portuguese populations. Still, while Azorean populations are intermixed, there appears to be a gradual distancing from Sesimbra (mainland Portugal) to Ilhéu de Cima (Porto Santo, Madeira) and finally to the Azores (Figure 3A). A low percentage of diversity (5% by coordinate 1, 3.59% by coordinate 2) is explained by the PCoA analysis, potentially resulting from the high diversity and evolutionary divergence of GBAS data within and between the regions sampled. While the number of alleles per marker is relatively high (mean of 148 per marker), the number of different alleles with a frequency higher than 5% is very low compared to the total amount of alleles (Table 2). Additionally, when the number of alleles is analyzed per population, it appears that more than half of these are private (Table 2). The small number of shared alleles among the analyzed markers is a likely indication of the latter not being informative.

The DAPC analysis revealed a similar pattern to the one inferred by the PCoA (Figure 3B,C). The Sesimbra (Portugal) population was found to differentiate most from the others (Figure 3B). The remaining populations mostly cluster with each other (Figure 3C).

**FIGURE 4:**
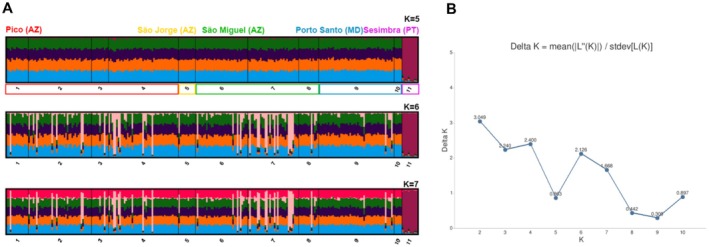
(A) Structure analysis showing the 11 analyzed populations as follows: 1—Calheta, Pico Island, Azores; 2—Fonte, Pico Island, Azores; 3—Furna, Pico Island, Azores; 4—S. Caetano, Pico Island, Azores; 5—Morro de Enseada, São Jorge Island, Azores; 6—Dori, São Miguel Island, Azores; 7—Ilhéu de Vila Franca, São Miguel Island, Azores; 8—Lagoa, São Miguel Island, Azores; 9—Ilhéu de Cima, Porto Santo Island, Madeira; 10—Porto, Porto Santo Island, Madeira; 11—Sesimbra, Portugal. (B) Delta *K*‐values; *K* = 2 inferred as optimal. *K* = 5, 6, 7 were displayed instead of the previous *K* (2, 3, 4) as the latter only showed horizontal lines and were thus not inferred as descriptive (B).

The STRUCTURE analysis revealed an optimal delta‐K of 2. Although only K‐5 and K‐7 showed some clustering, suggesting that no clear structuring can be found between the NE Atlantic archipelagos of Madeira and the Azores, only populations from Sesimbra are distinct from the remaining (Figure 4). BayesAss analysis revealed that most populations have an average of 2% of migrants from all the populations sampled within the NE Atlantic (Table S4). The Sesimbra population was found to contribute the most in terms of migrants to the population in Dori (São Miguel Island, Azores) with a total of 11%.

BOTTLENECK v 1.2.02 showed no significant deviation of observed heterozygosity from the expected under mutation‐drift equilibrium (Table 7). We thus do not infer any sign of a bottleneck within the analyzed populations of *E. castanea* .

**TABLE 7:** Bottleneck analysis computed for the studied populations.

Population	Mean N	Mean k	Mean *H*_e_	*p*	SD
Calheta, PIX, AZO	21.50	12.33	0.90103	0.371610	0.263398
Fonte, PIX, AZO	62.17	29.75	0.91932	0.143908	0.018464
Furna, PIX, AZO	17.00	9.75	0.88222	0.544715	0.194773
São Caetano, PIX, AZO	68.50	33.75	0.91457	0.164571	0.000000
Morro da Enseada, SJZ, AZO	16.67	9.75	0.90352	0.613628	0.197802
Dori, SMG, AZO	54.17	26.33	0.91271	0.318760	0.000000
Ilhéu V. Franca, SMG, AZO	52.67	23.08	0.85239	0.059939	0.000000
Lagoa, SMG, AZO	19.50	10.58	0.85763	0.227080	0.488520
Ilhéu de Cima, PXO, MAD	69.67	33.67	0.93959	0.170469	0.002799
Porto, PS, MAD	5.17	3.42	0.69663	0.406187	0.089819
Sesimbra, POR	9.17	3.08	0.44289	0.055957	0.108694

*Note:* The observed heterozygosity (*H*_e_) obtained from the number of alleles (*k*) sample size (*N*) of each population, and under the assumptions of mutation‐drift equilibrium is shown. The *p*‐value of the observed heterozygosity and standard deviation under the Infinite Allele Model is also shown. For populations' abbreviations, please see the legend of Table 1.

### 3.2 mtDNA COI Data

**FIGURE 5:**
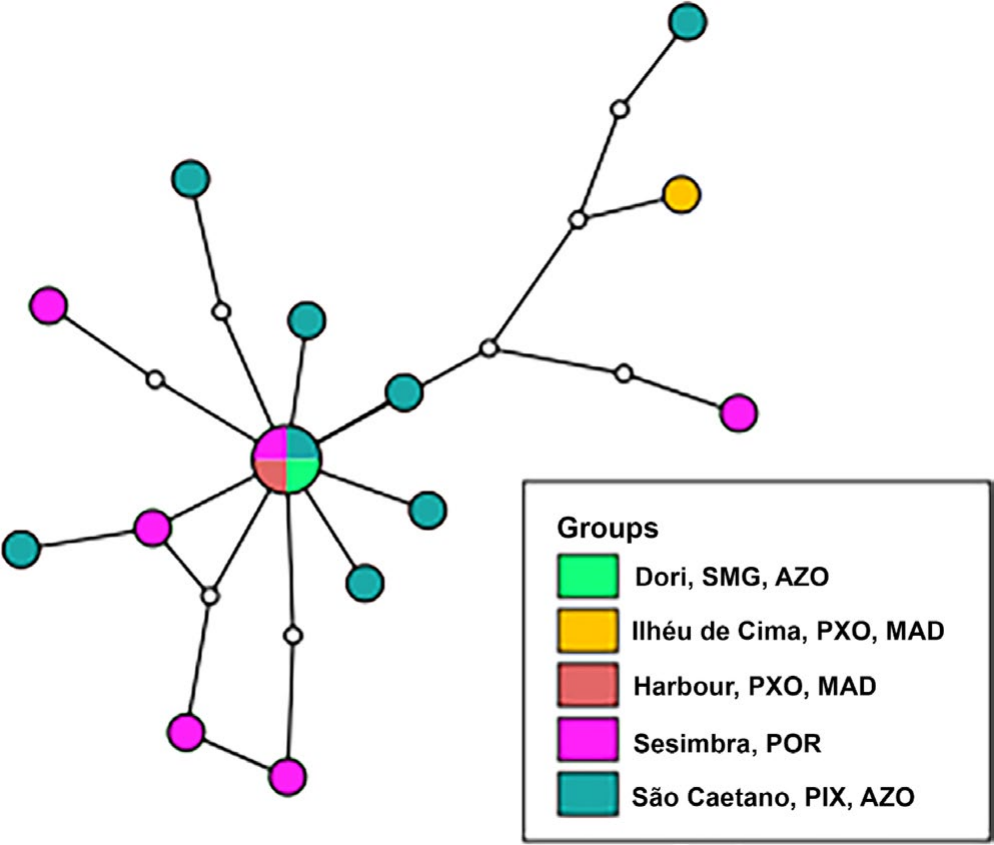
Distribution of mitochondrial haplotypes in the NE Atlantic of *Ervilia castanea* , based on 17 COI sequences, resulting in 14 haplotypes: One haplotype is shared between four individuals (larger circle), whereas the remaining 13 are single occurrences. Geographical representation of islands/localities color coded. For abbreviations of sites or regions, please see legend of Table 1.

The COI dataset comprised a total of 17 sequences from Sesimbra (mainland Portugal), Madeira, and Azores (Table S2, Additional Files). The TCS analysis showed no geographical or genetic structure among individuals or between the mainland and Atlantic archipelagos (Figure 5). The program estimated the presence of several missing haplotypes, indicating insufficient sampling of the COI dataset.

## 4 Discussion

### 4.1 Genetic Phenomena Affecting Multilocus Analysis

Many GBAS markers in our study failed amplification. Indeed, out of the 50 designed, only 25 passed the quality control steps, and of these, only 12 had < 60% missing data. Moreover, the amplicon length distribution plots showed that at least three loci (Figure S1) had duplicated regions, and only four were informative of the genetic patterns retrieved. This lack of resolution may have biological or technical causes and is likely related to duplication, a high prevalence of null alleles (mean of 18% null alleles per locus and population), and hypervariability of screened genomic regions, which might also cause deviations from HWE. Null alleles are part of the very high allelic diversity found in the dataset, increasing the likelihood of divergence between alleles at primer binding sites. HWE deviation may also be related to gene duplication. If duplicated markers are retained in the dataset, the genotyping of either or both paralog loci can inflate the estimates of null alleles and HWE deviations (see Carlsson 2008; Curto et al. 2019; Turini et al. 2014 for a review).

Although our dataset does not allow for the quantification of duplication events, which would require whole‐genome datasets, the pattern of duplication found in the marker plots, together with high levels of null alleles, suggests that such mechanisms are widespread among the loci of *E. castanea* . This may be explained by a relatively recent polyploidization event within an *E. castanea* lineage. Polyploidy is more common in plants than in animals (Mable 2004), but still plays an important evolutionary role in nearly every group of organisms (Allendorf et al. 2015). Chromosomal rearrangements and recombination related to polyploidy may play a significant role in speciation (Allendorf et al. 2015). Even though reproductive isolation mechanisms remain mostly unknown, most marine bivalves are broadcast spawners, and chromosomal rearrangements may serve as species‐selective pre‐reproductive barriers (Wang and Guo 2004). At least one genome duplication event has occurred in marine bivalves (Wang and Guo 2004), suggesting that polyploidy might be a common phenomenon in this clade. One of the most successfully dispersed marine bivalve genera, *Lasea* (Brown, 1827), is composed of highly polyploid clonal lineages, many of which display supernumerary chromosomes (Foighil and Thiriot‐Quiévreux 1999). The possible existence of such genome duplication events in *E. castanea* requires further investigation.

Another noteworthy pattern arising from the WAI dataset of *E. castanea* is the high diversity encountered within the sampled markers (Table 2) on a level that was called hyperdiversity in an example from gastropods (Fourdrilis et al. 2016; Fourdrilis and Backeljau 2019). Nuclear hyperdiversity is defined by allelic divergence exceeding 5%, and although such patterns are most commonly found in viruses and bacteria, the lack of evidence among eukaryotic species may be compounded by a bias toward the study of organisms with large body sizes (> 1 mm) (Cutter et al. 2013). Such high levels of nucleotide variability resulted in an inflation in the number of alleles, especially when WAI is considered. The increasing implementation of high‐throughput next‐generation sequencing platforms facilitates the investigation of small‐size organisms. Emerging data show that multicellular eukaryotic organisms can also harbor exceptional levels of molecular polymorphism, caused by very large population sizes that allow the accumulation of diversity and a reduced effect of genetic drift in removing alleles (Cutter et al. 2013). Besides high population census and effective population sizes, other ecological determinants of hyperdiversity include small individual size, the ability to exploit dense or widespread resources, and consequent rapid development (Cutter et al. 2013). Such factors, specifically high dispersal propensity and large geographic ranges, are inherent to many shallow‐water marine broadcast spawners (Jiang and Smith 2005; Therriault and Herborg 2008) and appear to be shared by *E. castanea*, likely explaining the high allelic diversity in this species.

Nucleotide hyperdiversity leads to a lack of shared alleles and a large proportion of singletons within the sampled populations. The information available to determine genetic structure and diversity is then low, as it depends on the comparison of allele frequencies among populations. In our analysis, many markers had a high number of alleles, most of which were private within a population, with many unique to a single individual and not informative for an allele frequency‐based analysis. Based on our data, it is not possible to determine whether the retrieved patterns are a consequence of relatively restricted gene flow in large populations or, rather, due to a high proportion of colonizers with abnormally high mutation rates, as described in Robalo et al. (2020). However, the fact that not all populations show equal levels of diversity indicates that intrinsic traits, such as mutation rate, alone cannot explain the hypervariability of the loci. For example, the population of Sesimbra showed a much lower number of alleles and genetic diversity than insular populations. As discussed below, this population is ecologically more stable and isolated than the populations found on the islands, indicating that demographic and ecological factors have to play a role in hyperdiversity. In each recruitment event, a new combination of this incredibly diverse pool of alleles will constitute the new generation, rendering its genetic diversity as high as that of the adult population. This contrasts with what would be expected from the ‘sweepstakes effect’, whereby a small proportion of the gene pool contributes to the recruitment of the population, thus promoting reduced genetic diversity in the following generations (Robalo et al. 2020). It is thus critical to incorporate such mutation models into population genetic analyses to achieve appropriate interpretations of the demographic history of hyperdiverse species. These organisms represent models for exploring a variety of evolutionary issues, from genome complexity to modes of adaptation, mutational dynamics, and fine‐scale inference of sequence function (Cutter et al. 2013). This has not yet been achieved with multilocus analysis of genotype markers. The increased use of sequence‐based markers, such as the GBAS method, holds promise that more examples of hyperdiversity, which remain undetected with indirect genotype information, such as length polymorphism in classical microsatellite analysis or single SNPs, will be found. Finally, hyperdiversity caused by large population sizes is also likely to result in a high prevalence of null alleles, as well as reduced amplification success of markers during optimisation.

### 4.2 Differentiation of *Ervilia castanea* in the Archipelagos

The above‐mentioned results additionally constrain the breadth of our discussion and advise a cautious interpretation of the resulting genetic patterns. Nevertheless, what stands out is the relative differentiation between the mainland population and the sampled archipelagos. Although only one mainland population was analyzed, hindering detailed interpretations, it was the only population not deviating from HWE (Table 3), suggesting that some specific evolutionary process is acting on the insular populations. Besides what has been previously described (i.e., Wahlund effect, inbreeding or null alleles), such patterns might alternatively be a consequence of the effects of differential demographic histories of the insular populations. *Ervilia castanea* is found in high abundance in sandy habitats in the Azores, which suffer significant temporal and physical disturbances, potentially causing cyclical population extirpations during interglacial/glacial transitions (Ávila, Madeira, Da Silva, et al. 2008). If insular populations are experiencing environmental pressure at a higher and more frequent pace compared to the mainland, they might undergo a higher turnover than mainland populations. Due to the high dispersal ability of this species, individuals originating from other populations would constantly replenish newly vacant habitats. Due to the stability of the mainland habitat (Ávila et al. 2019), this process may be less prevalent. Our results suggest constant gene flow and connectivity between mainland and insular populations in the NE Atlantic, which might be causative of high population sizes and high allelic numbers and variation, potentially masking the signature of bottlenecks (undetected with our BOTTLENECK analysis; Table 7) and extirpation events in the genetic signal of insular populations.

The PCoA (Figure 3A), DAPC (Figure 3B,C) and STRUCTURE (Figure 4) analyses show no clear differentiation between the sampled archipelagos, which cluster together and only differentiate from the mainland population (Sesimbra). The COI dataset also shows no structure among populations, although this can be partially related to the low number of samples included (17). The BayesAss analysis of microsatellite data shows an equal percentage of migrating individuals contributing to and from the genetic pools of the sampled populations, with Sesimbra being the greater source of migrants (11%) to the populations of Dori (São Miguel, Azores). Although no definite conclusions can be inferred hereby due to the underrepresentation of mainland populations, other studies suggest that the Azores has served as a recipient of gene flow from ancient and current West‐European populations (Ávila 2000a, 2000b, 2005; 2013; Sala et al. 2013; Baptista et al. 2021), potentially defining a pattern also at work within the current system. Shared species assemblages between NE Atlantic archipelagos and the western European shores have been found for other marine clades such as Porifera, Cnidaria, Annelida, Arthropoda, and Echinodermata, despite this pattern not being consistent with the present sea‐surface circulation in the North Atlantic (Ávila, Madeira, et al. 2009; Freitas et al. 2019). Since the progressive closure of the Isthmus of Panama (13—2.7 Ma), the Gulf Stream has been the major hydrographical feature influencing the climate in the Azores region, increasing in strength, velocity, and volume during glacial periods (O'Dea et al. 2016). As a consequence, the probability of the arrival of western Atlantic species, i.e., of warm‐water Caribbean species, to the Azores would be higher during glacial periods. However, environmental conditions in the Azores during glacial periods, i.e., optimal sea surface temperatures (SST), required for the establishment of viable, reproductive populations, may not match the ones from where the dispersing larvae originated. This mismatch would probably result in failed colonization events following long‐distance dispersal opportunities or, in the best‐case scenario, in the formation of pseudo‐populations, the maintenance of which would rely solely on the regular arrival of new colonizers (Ávila, Da Marques Silva, et al. 2009). Based on solid data from the fossil record, Ávila and colleagues suggested in a series of papers that during the final phase of glacial termination II (the short period of about 6000 y that marks the end of the MIS 6 glacial period and the beginning of the MIS 5e interglacial period), or shortly after the emplacement of the MIS 5e, short‐lived oceanic currents, different from those observed today, might have been established. These currents could have facilitated the arrival of tropical and temperate species from Cabo Verde Archipelago and the eastern Atlantic shores, respectively, to the Azores (Ávila, Da Marques Silva, et al. 2009; Ávila, Madeira, et al. 2009; Ávila, Melo, Silva, et al. 2015; Ávila, Ramalho, Habermann, et al. 2015; Ávila et al. 2019; Melo et al. 2022a, 2022b). Despite the relatively limited lifetime of such currents, shared environmental conditions within NE Atlantic continental and insular locations during the interglacial MIS 5e period would have conferred an optimal setting for the establishment of viable and successful populations. When the present oceanographic regime was reestablished, it might have become easier for islands to exchange propagules among their populations than with the mainland. At the same time, the geographical proximity between the archipelagos and Europe would still have enabled gene flow between populations, despite a predominantly eastward circulation regime (Ávila, Da Marques Silva, et al. 2009), a pattern supported by the overall admixture of insular populations and some degree of distance between islands and the continent, as revealed by SSR analyses.

Throughout the quaternary period, the alteration of oceanographic conditions triggered by glacial/interglacial cycles and the consequent exposure/submersion of oceanic seamounts was crucial in determining either opportunities or barriers for the dispersal of marine benthic fauna within the NE Atlantic (Ávila 2013). During glacial periods, for example, as shallow seamounts became islands, functioning as stepping stones during times of low sea levels (e.g., Ampère, Seine or Ormonde seamount), the dispersal of mainland species toward the islands was further facilitated (Ávila and Malaquias 2003). The geographic isolation of Madeira, for instance, was estimated to have decreased by as much as 53.2% (Freitas et al. 2019). Such events might have enabled the dispersal of *E. castanea* from the mainland to Madeira. During the Last Interglacial, Madeira and the Azores biogeographically clustered together and were set apart from another cluster formed by the Canary Islands and Cabo Verde (Melo et al. 2023). In contrast, recent biogeographical patterns of extant marine shallow‐water species indicate Madeira, Selvagens and the Canary Islands as the core of Macaronesia, forming the so‐called Webbnesia ecoregion. Despite the Azores being considered a separate ecoregion today, biogeographic affinities have been found for some widely dispersing groups, such as coastal fishes, echinoderms, and macroalgae, indicating this archipelago as a close sister group to Webbnesia (Freitas et al. 2019). The combination of *E. castanea* 's high dispersal ability, the ephemeral nature of the habitats it explores and occupies, and the current and past environmental conditions that favor connectivity within the NE Atlantic archipelagos and, to some degree, with the mainland has contributed to the retrieved population genetic patterns.

## 5 Conclusions

The current study serves as a primary insight into the potential evolutionary mechanisms affecting species inhabiting the sandy habitats of oceanic islands. The patterns retrieved suggest *E. castanea* populations continuously exchange migrants throughout the NE Atlantic. Despite the evolutionary and ecological dynamism of oceanic islands, which results in sandy habitats being mostly devoid of life, the high dispersal ability of *E. castanea* might fuel constant recolonization of these environments, thus hindering the effects of potential population bottlenecks or even extirpation/local disappearance. As high larval inflow replenishes newly vacant habitats and increases connectivity between oceanic islands, individuals within the same populations might be more likely to have originated from distant populations than from local offspring. Structural genetic differentiation appears between populations of oceanic islands and the nearest continental shores. However, only one population from the mainland was sampled, limiting any generalization to the other related sandy habitats. Nonetheless, such patterns should inspire further population studies comparing oceanic and continental soft‐bottom shores.

Future investigations should focus on understanding whether such disparities remain when larger sample sizes are included in the analysis. Is the environment in oceanic islands selecting for species with good dispersal abilities, which would eventually differentiate from mainland populations living in relatively more favorable environmental conditions? Are there any post‐settlement reproductive barriers between insular and continental sandy habitats? By answering these questions, further insights can be brought into the understanding of the uniqueness of ecological interactions at play in the sandy habitats of oceanic islands. Such understanding is essential, as these habitats might be of primary importance in sustaining the overall intraspecific biodiversity of marine populations worldwide. Especially at times when anthropogenic climate change poses serious threats to global diversity patterns, such studies may reveal the crucial role of oceanic islands in supporting biodiversity and enhancing the resilience of a species facing environmental threats.

Another important factor arising from this study is the hypervariability of the alleles studied together with a high presence of null alleles. Deviations from HWE and the high presence of null alleles seem to be common in marine invertebrates and were observed in marine gastropods (Panova et al. 2008; Holborn et al. 2015; López‐Márquez et al. 2016) and bivalves (Hedgecock et al. 2004; Rico et al. 2017; Hargrove et al. 2015; Chiesa et al. 2016). Investigating the genetic pattern of Wedge Clam (*Donax trunculus*) across the Mediterranean and the Atlantic Ocean, Rico et al. (2017) found a significant excess of homozygotes, deviations from HWE together with a high frequency of null alleles, to the point that only three (two of which had a high frequency of null alleles) out of 16 markers were informative of the patterns retrieved. The fact that panmixia was detected where no apparent barriers to gene flow persisted, while a well‐defined structure for populations separated by a distinct biogeographic barrier and that null alleles correction through FreeNa had no effect in the F_ST_ estimates, led Rico et al. (2017) to conclude that even when a large frequency of null alleles is present, the genetic structure patterns of populations can be analyzed although levels of genetic diversity are most likely underestimated.

Although our dataset does not allow for further genomic testing, thus highlighting the assumptive nature of our discussion, the similar pattern retrieved to the above‐mentioned studies calls for directed investigations within the evolutionary dynamics of marine mollusks. As new and fast‐evolving NGS methodologies are exposing the virtually unlimited number of polymorphic microsatellites across a range of taxa (Šarhanová et al. 2018 and references therein), there is the need to jointly evolve new genetic reference systems that take into consideration less commonly studied organisms such as marine invertebrates. Future research, for example, should feature markers that are less sensitive to hypervariability and null alleles so that deeper genetic patterns can be retrieved.

## Author Contributions

**Livia Sinigaglia:** conceptualization (equal), data curation (equal), formal analysis (equal), investigation (equal), methodology (equal), visualization (equal), writing – original draft (equal). **António Múrias Santos:** funding acquisition (equal), project administration (equal), resources (equal), software (equal), supervision (equal), validation (equal), writing – review and editing (equal). **Harald Meimberg:** conceptualization (equal), funding acquisition (equal), project administration (equal), resources (equal), software (equal), supervision (equal), validation (equal), writing – review and editing (equal). **Manuel Curto:** formal analysis (equal), methodology (equal), project administration (equal), resources (equal), software (equal), supervision (equal), validation (equal), writing – review and editing (equal). **Sérgio P. Ávila:** conceptualization (equal), funding acquisition (equal), project administration (equal), resources (equal), supervision (equal), validation (equal), writing – review and editing (equal). **Lara Baptista:** investigation (equal), validation (equal), writing – review and editing (equal). **Patrícia Madeira:** investigation (equal), writing – review and editing (equal). **Thapasya Vijayan:** investigation (equal), writing – review and editing (equal).

## Conflicts of Interest

The authors declare no conflicts of interest.

## Supporting information


Appendix S1.

## Data Availability

The datasets generated and/or analyzed during the current study are available in the Genbank repository, BioProject ID: PRJNA1137574.
