# Supplementary material for: Population Dynamics of Ervilia castanea (Montagu, 1803) Hints at Evolutionary Processes Shaping North‐East Atlantic Insular Sandy Habitats
Source: Ecol Evol. 2025 Jun 3;15(6):e71267. doi: 10.1002/ece3.71267 (PMC12130739; doi:10.1002/ece3.71267)
Supplement: Supplementary file 1 — Appendix S1. [file ECE3-15-e71267-s001.docx]

**Supplementary Material**

**Table S1** *-* **Complete list of all SSR primers designed in this study**. Includes sequence of forward and reverse primers (5’-3’), the repetition motif, and number of repeats in the original sequence used for the primer design, the primer-mix in which it was included for the Multiplex PCR, and Information regarding failed primers.

| **Primer/Loci** | **Repetition motif** | **Forward (5’-3’)** | **Reverse (5’-3’)** | **Mix** |
| --- | --- | --- | --- | --- |
| SPEC4 | 6(TAGA) | AAATATTAAGAAGTGACAGATGAG | ACAAAGTGTTATGATCGATTCT | PM1 |
| SPEC8 | 4(ATATG) | CAAACAAGTGTACTAGAAAGTATG | TTTGTACTACATAACAGCATATGA | PM1 |
| SPEC10 | 4(ATATT) | TGTGAATGCTTTACCAAACA | ATATGCACGGGTGTCTTTTT | PM1 |
| SPEC21 | ATATG | AAGTCCCCATTCAGTTGATC | AGAAGACAAAGTTAAAAGTATCG | PM1 |
| SPEC29 | 6(TGTT) | CTGTGGATGTGTGACATACA | GCCCCTGTGATGGTAAATAA | PM1 |
| SPEC52 | 6(AGC) | CTCACCATCTGTCTGCTCCG | GCAAATGTTCCGGTCACAGG | PM1 |
| SPEC55 | 9(TAA) | CTCGTCACAGCATTTAAGAAG | CCAATGTGTTTTTCTCAGACT | PM1 |
| SPEC57 | 7(ATC) | ATGAAGTCAGCGAGGAGTGC | GAGGGCGTGACATTAATGGC | PM1 |
| SPEC58 | 6(ATT) | AAACCCTCCCAATGACGGAC | AGGCCCACATGGAAGATTGG | PM1 |
| EV2 | 4(TTGAA) | CACTGTTCGCTTGTTTGTAT | CCTTCAGATCCTCAGACTA | PM1 |
| EV7 | 6(ATATG) | CCTTTGTCACGAGTGTTACT | CCCAAGTGTCGAAACTGAAT | PM1 |
| EV8 | 5(AATTG) | CCATTCAATTCGCAGTCATT | TGGGATTGTGTTGTCCATTA | PM1 |
| SPEC6 | ATTGT | TCCAATTTTTCTTCTCCGTCTA | TCTAAGGCTTCAAACATTCCTA | PM2 |
| SPEC7 | TCAAG | CCAAAAACGATCCCCTTTTT | TGTTTGGAGGTCTCTCACTA | PM2 |
| SPEC9 | ATATC | GCTTATGTCACTTCTACTATCG | GATATAAGCCCGCTCGAATT | PM2 |
| SPEC17 | AAAG | AGCATTCATGTGACATGTTT | CGGCAAGTGACTTTGACATA | PM2 |
| SPEC23 | 5(ATGA) | CTCTCCCAACGAGTTTATGA | ACACCTGTACAAGACAATGT | PM2 |
| SPEC24 | 6(CAAT) | AGAGATCTAACCACAGCTAAT | TTTCGACAAGCCCTCTTATC | PM2 |
| SPEC32 | 6(TATGA) | ACAATTGCTTCCCTTTATCT | AGGGTCGCTAAAAGTGTTTA | PM2 |
| SPEC36 | 4(CTGAA) | TACACGGCTGATTTTCATTG | ACCTTCCGATAACGACTTTT | PM2 |
| SPEC47 | 4(AGAAC) | ACGTTAAGGTCACAGCAATA | TCCAATTAGTACAAAAGGTATGT | PM2 |
| SPEC48 | 4(ATTGA) | GCTTTCAATGCAGTGGTAAT | ACTAGAAGCGCCAAATCATC | PM2 |
| SPEC49 | 4(AGTTT) | TATCTCTCCCCACAGATGTT | TCATTTTGAGCAATATAGTATGTT | PM2 |
| SPEC56 | 6(TCA) | GTCGCCAATGACTGTTCACA | GCAGTGAAGGCAGCCTGTAA | PM2 |
| EV1 | 6(TATGA) | ACAATTGCTTCCCTTTATCT | AGGGTCGCTAAAAGTGTTTA | PM2 |

**Table S2.** Sample Size (N), No. Alleles (Na), No. Effective Alleles (Ne), Information Index (I), Observed Heterozygosity (Ho), Expected (He) and Unbiased Expected Heterozygosity (uHe), and Fixation Index (F) per locus and population sampled of *E. castanea.*

| Population | Locus | N | Na | Ne | I | Ho | He | uHe | F |
| --- | --- | --- | --- | --- | --- | --- | --- | --- | --- |
| Calheta, PIX, AZO | **SP2EC6_ATTGT** | 13 | 19.000 | 14.696 | 2.832 | 0.615 | 0.932 | 0.969 | 0.340 |
|  | **Ev1_TATGA** | 13 | 18.000 | 15.364 | 2.811 | 0.462 | 0.935 | 0.972 | 0.506 |
|  | **32SP2EC_TATGA** | 13 | 18.000 | 15.364 | 2.811 | 0.462 | 0.935 | 0.972 | 0.506 |
|  | **SP2EC9_ATATC** | 13 | 11.000 | 7.682 | 2.205 | 0.154 | 0.870 | 0.905 | 0.823 |
|  | **23SP2EC_ATGA** | 12 | 12.000 | 4.174 | 1.963 | 0.583 | 0.760 | 0.793 | 0.233 |
|  | **29SP2EC_TGTT** | 11 | 12.000 | 6.722 | 2.220 | 0.455 | 0.851 | 0.892 | 0.466 |
|  | **SP2EC57_3mer** | 11 | 14.000 | 12.737 | 2.587 | 0.273 | 0.921 | 0.965 | 0.704 |
|  | **48SP2EC_ATTGA** | 8 | 11.000 | 9.846 | 2.339 | 0.375 | 0.898 | 0.958 | 0.583 |
|  | **SP2EC7_TCAAG** | 8 | 9.000 | 7.111 | 2.079 | 0.250 | 0.859 | 0.917 | 0.709 |
|  | **Ev2_TTGAA** | 11 | 5.000 | 2.262 | 1.136 | 0.182 | 0.558 | 0.584 | 0.674 |
|  | **SP2EC8_ATATG** | 9 | 12.000 | 10.800 | 2.428 | 0.333 | 0.907 | 0.961 | 0.633 |
|  | **Ev8_AATTG** | 7 | 7.000 | 7.000 | 1.946 | 0.000 | 0.857 | 0.923 | 1.000 |
| Fonte, PIX, AZO | **SP2EC6_ATTGT** | 37 | 38.000 | 18.013 | 3.302 | 0.622 | 0.944 | 0.957 | 0.342 |
|  | **Ev1_TATGA** | 40 | 52.000 | 30.476 | 3.758 | 0.575 | 0.967 | 0.979 | 0.405 |
|  | **32SP2EC_TATGA** | 40 | 51.000 | 27.826 | 3.717 | 0.550 | 0.964 | 0.976 | 0.429 |
|  | **SP2EC9_ATATC** | 36 | 34.000 | 20.736 | 3.312 | 0.306 | 0.952 | 0.965 | 0.679 |
|  | **23SP2EC_ATGA** | 38 | 17.000 | 2.908 | 1.739 | 0.421 | 0.656 | 0.665 | 0.358 |
|  | **29SP2EC_TGTT** | 26 | 26.000 | 11.361 | 2.927 | 0.462 | 0.912 | 0.930 | 0.494 |
|  | **SP2EC57_3mer** | 31 | 32.000 | 25.973 | 3.372 | 0.129 | 0.961 | 0.977 | 0.866 |
|  | **48SP2EC_ATTGA** | 26 | 22.000 | 11.757 | 2.795 | 0.308 | 0.915 | 0.933 | 0.664 |
|  | **SP2EC7_TCAAG** | 32 | 31.000 | 19.883 | 3.249 | 0.281 | 0.950 | 0.965 | 0.704 |
|  | **Ev2_TTGAA** | 32 | 12.000 | 3.385 | 1.708 | 0.313 | 0.705 | 0.716 | 0.556 |
|  | **SP2EC8_ATATG** | 24 | 26.000 | 25.043 | 3.236 | 0.083 | 0.960 | 0.980 | 0.913 |
|  | **Ev8_AATTG** | 19 | 21.000 | 19.000 | 2.990 | 0.211 | 0.947 | 0.973 | 0.778 |
| Furna, PIX, AZO | **SP2EC6_ATTGT** | 10 | 13.000 | 10.000 | 2.441 | 0.600 | 0.900 | 0.947 | 0.333 |
|  | **Ev1_TATGA** | 10 | 16.000 | 14.286 | 2.718 | 0.700 | 0.930 | 0.979 | 0.247 |
|  | **32SP2EC_TATGA** | 10 | 16.000 | 14.286 | 2.718 | 0.700 | 0.930 | 0.979 | 0.247 |
|  | **SP2EC9_ATATC** | 10 | 9.000 | 7.692 | 2.112 | 0.200 | 0.870 | 0.916 | 0.770 |
|  | **23SP2EC_ATGA** | 10 | 7.000 | 2.247 | 1.259 | 0.300 | 0.555 | 0.584 | 0.459 |
|  | **29SP2EC_TGTT** | 8 | 9.000 | 8.533 | 2.166 | 0.125 | 0.883 | 0.942 | 0.858 |
|  | **SP2EC57_3mer** | 10 | 10.000 | 7.692 | 2.177 | 0.200 | 0.870 | 0.916 | 0.770 |
|  | **48SP2EC_ATTGA** | 8 | 7.000 | 5.565 | 1.820 | 0.125 | 0.820 | 0.875 | 0.848 |
| Population | Locus | N | Na | Ne | I | Ho | He | uHe | F |
|  | **SP2EC7_TCAAG** | 9 | 11.000 | 10.125 | 2.351 | 0.222 | 0.901 | 0.954 | 0.753 |
|  | **Ev2_TTGAA** | 8 | 4.000 | 2.246 | 1.041 | 0.250 | 0.555 | 0.592 | 0.549 |
|  | **SP2EC8_ATATG** | 8 | 10.000 | 9.143 | 2.253 | 0.250 | 0.891 | 0.950 | 0.719 |
|  | **Ev8_AATTG** | 9 | 11.000 | 10.125 | 2.351 | 0.222 | 0.901 | 0.954 | 0.753 |
| São Caetano, PIX, AZO | **SP2EC6_ATTGT** | 43 | 45.000 | 10.159 | 3.222 | 0.674 | 0.902 | 0.912 | 0.252 |
|  | **Ev1_TATGA** | 43 | 58.000 | 26.414 | 3.793 | 0.605 | 0.962 | 0.973 | 0.372 |
|  | **32SP2EC_TATGA** | 43 | 59.000 | 26.604 | 3.809 | 0.628 | 0.962 | 0.974 | 0.348 |
|  | **SP2EC9_ATATC** | 37 | 25.000 | 6.967 | 2.663 | 0.216 | 0.856 | 0.868 | 0.748 |
|  | **23SP2EC_ATGA** | 38 | 21.000 | 3.877 | 2.113 | 0.526 | 0.742 | 0.752 | 0.291 |
|  | **29SP2EC_TGTT** | 32 | 33.000 | 15.754 | 3.184 | 0.469 | 0.937 | 0.951 | 0.499 |
|  | **SP2EC57_3mer** | 31 | 35.000 | 28.687 | 3.461 | 0.226 | 0.965 | 0.981 | 0.766 |
|  | **48SP2EC_ATTGA** | 28 | 23.000 | 13.067 | 2.872 | 0.250 | 0.923 | 0.940 | 0.729 |
|  | **SP2EC7_TCAAG** | 26 | 29.000 | 24.582 | 3.290 | 0.231 | 0.959 | 0.978 | 0.759 |
|  | **Ev2_TTGAA** | 37 | 16.000 | 2.986 | 1.722 | 0.324 | 0.665 | 0.674 | 0.512 |
|  | **SP2EC8_ATATG** | 30 | 35.000 | 32.727 | 3.517 | 0.167 | 0.969 | 0.986 | 0.828 |
|  | **Ev8_AATTG** | 29 | 30.000 | 29.000 | 3.382 | 0.069 | 0.966 | 0.982 | 0.929 |
| Morro da Enseada, SJZ, AZO | **SP2EC6_ATTGT** | 11 | 15.000 | 8.345 | 2.453 | 0.818 | 0.880 | 0.922 | 0.070 |
|  | **Ev1_TATGA** | 10 | 14.000 | 11.765 | 2.554 | 0.500 | 0.915 | 0.963 | 0.454 |
|  | **32SP2EC_TATGA** | 10 | 14.000 | 11.765 | 2.554 | 0.500 | 0.915 | 0.963 | 0.454 |
|  | **SP2EC9_ATATC** | 11 | 14.000 | 12.100 | 2.563 | 0.455 | 0.917 | 0.961 | 0.505 |
|  | **23SP2EC_ATGA** | 11 | 7.000 | 3.143 | 1.463 | 0.364 | 0.682 | 0.714 | 0.467 |
|  | **29SP2EC_TGTT** | 9 | 11.000 | 10.125 | 2.351 | 0.222 | 0.901 | 0.954 | 0.753 |
|  | **SP2EC57_3mer** | 10 | 11.000 | 9.091 | 2.303 | 0.200 | 0.890 | 0.937 | 0.775 |
|  | **48SP2EC_ATTGA** | 10 | 7.000 | 4.545 | 1.748 | 0.000 | 0.780 | 0.821 | 1.000 |
|  | **SP2EC7_TCAAG** | 7 | 8.000 | 6.125 | 1.946 | 0.429 | 0.837 | 0.901 | 0.488 |
|  | **Ev2_TTGAA** | 10 | 8.000 | 3.922 | 1.704 | 0.500 | 0.745 | 0.784 | 0.329 |
|  | **SP2EC8_ATATG** | 7 | 8.000 | 7.538 | 2.045 | 0.143 | 0.867 | 0.934 | 0.835 |
|  | **Ev8_AATTG** | 5 | 6.000 | 5.556 | 1.748 | 0.200 | 0.820 | 0.911 | 0.756 |
| Dori, SMG, AZO | **SP2EC6_ATTGT** | 32 | 33.000 | 10.240 | 3.035 | 0.625 | 0.902 | 0.917 | 0.307 |
|  | **Ev1_TATGA** | 34 | 45.000 | 17.126 | 3.472 | 0.676 | 0.942 | 0.956 | 0.282 |
|  | **32SP2EC_TATGA** | 34 | 45.000 | 17.126 | 3.472 | 0.676 | 0.942 | 0.956 | 0.282 |
|  | **SP2EC9_ATATC** | 31 | 18.000 | 5.555 | 2.304 | 0.258 | 0.820 | 0.833 | 0.685 |
|  | **23SP2EC_ATGA** | 33 | 25.000 | 5.935 | 2.517 | 0.545 | 0.831 | 0.844 | 0.344 |
|  | **29SP2EC_TGTT** | 29 | 30.000 | 19.788 | 3.210 | 0.310 | 0.949 | 0.966 | 0.673 |
|  | **SP2EC57_3mer** | 25 | 23.000 | 16.892 | 2.988 | 0.280 | 0.941 | 0.960 | 0.702 |
| Population | Locus | N | Na | Ne | I | Ho | He | uHe | F |
|  | **48SP2EC_ATTGA** | 19 | 16.000 | 12.237 | 2.641 | 0.316 | 0.918 | 0.943 | 0.656 |
|  | **SP2EC7_TCAAG** | 28 | 23.000 | 17.231 | 3.000 | 0.107 | 0.942 | 0.959 | 0.886 |
|  | **Ev2_TTGAA** | 23 | 12.000 | 2.692 | 1.559 | 0.348 | 0.629 | 0.643 | 0.447 |
|  | **SP2EC8_ATATG** | 25 | 30.000 | 27.778 | 3.358 | 0.280 | 0.964 | 0.984 | 0.710 |
|  | **Ev8_AATTG** | 23 | 25.000 | 22.511 | 3.166 | 0.130 | 0.956 | 0.977 | 0.864 |
| Ilhéu V. Franca, SMG, AZO | **SP2EC6_ATTGT** | 31 | 30.000 | 8.076 | 2.839 | 0.581 | 0.876 | 0.891 | 0.337 |
|  | **Ev1_TATGA** | 32 | 33.000 | 7.474 | 2.872 | 0.500 | 0.866 | 0.880 | 0.423 |
|  | **32SP2EC_TATGA** | 32 | 33.000 | 7.474 | 2.872 | 0.500 | 0.866 | 0.880 | 0.423 |
|  | **SP2EC9_ATATC** | 31 | 20.000 | 7.975 | 2.587 | 0.129 | 0.875 | 0.889 | 0.852 |
|  | **23SP2EC_ATGA** | 30 | 10.000 | 1.991 | 1.238 | 0.300 | 0.498 | 0.506 | 0.397 |
|  | **29SP2EC_TGTT** | 28 | 27.000 | 13.288 | 2.980 | 0.321 | 0.925 | 0.942 | 0.652 |
|  | **SP2EC57_3mer** | 23 | 25.000 | 14.694 | 2.967 | 0.391 | 0.932 | 0.953 | 0.580 |
|  | **48SP2EC_ATTGA** | 25 | 22.000 | 16.447 | 2.959 | 0.320 | 0.939 | 0.958 | 0.659 |
|  | **SP2EC7_TCAAG** | 25 | 26.000 | 20.492 | 3.136 | 0.360 | 0.951 | 0.971 | 0.622 |
|  | **Ev2_TTGAA** | 25 | 8.000 | 1.786 | 1.046 | 0.240 | 0.440 | 0.449 | 0.455 |
|  | **SP2EC8_ATATG** | 28 | 34.000 | 31.360 | 3.481 | 0.250 | 0.968 | 0.986 | 0.742 |
|  | **Ev8_AATTG** | 16 | 16.000 | 16.000 | 2.773 | 0.000 | 0.938 | 0.968 | 1.000 |
| Lagoa, SMG, AZO | **SP2EC6_ATTGT** | 11 | 13.000 | 5.902 | 2.209 | 0.818 | 0.831 | 0.870 | 0.015 |
|  | **Ev1_TATGA** | 11 | 15.000 | 13.444 | 2.650 | 0.545 | 0.926 | 0.970 | 0.411 |
|  | **32SP2EC_TATGA** | 11 | 15.000 | 13.444 | 2.650 | 0.545 | 0.926 | 0.970 | 0.411 |
|  | **SP2EC9_ATATC** | 12 | 14.000 | 10.286 | 2.496 | 0.333 | 0.903 | 0.942 | 0.631 |
|  | **23SP2EC_ATGA** | 11 | 8.000 | 2.086 | 1.245 | 0.545 | 0.521 | 0.545 | -0.048 |
|  | **29SP2EC_TGTT** | 10 | 13.000 | 10.526 | 2.458 | 0.500 | 0.905 | 0.953 | 0.448 |
|  | **SP2EC57_3mer** | 11 | 11.000 | 7.563 | 2.224 | 0.182 | 0.868 | 0.909 | 0.790 |
|  | **48SP2EC_ATTGA** | 10 | 11.000 | 8.696 | 2.276 | 0.400 | 0.885 | 0.932 | 0.548 |
|  | **SP2EC7_TCAAG** | 6 | 8.000 | 7.200 | 2.023 | 0.333 | 0.861 | 0.939 | 0.613 |
|  | **Ev2_TTGAA** | 12 | 5.000 | 1.907 | 0.998 | 0.083 | 0.476 | 0.496 | 0.825 |
|  | **SP2EC8_ATATG** | 10 | 13.000 | 11.765 | 2.511 | 0.300 | 0.915 | 0.963 | 0.672 |
|  | **Ev8_AATTG** | 10 | 10.000 | 10.000 | 2.303 | 0.000 | 0.900 | 0.947 | 1.000 |
| Ilhéu de Cima, PXO, MAD | **SP2EC6_ATTGT** | 45 | 31.000 | 10.438 | 2.899 | 0.467 | 0.904 | 0.914 | 0.484 |
|  | **Ev1_TATGA** | 47 | 58.000 | 29.258 | 3.823 | 0.532 | 0.966 | 0.976 | 0.449 |
|  | **32SP2EC_TATGA** | 47 | 60.000 | 32.014 | 3.876 | 0.574 | 0.969 | 0.979 | 0.407 |
|  | **SP2EC9_ATATC** | 42 | 26.000 | 10.167 | 2.833 | 0.095 | 0.902 | 0.913 | 0.894 |
|  | **23SP2EC_ATGA** | 30 | 20.000 | 4.787 | 2.290 | 0.267 | 0.791 | 0.805 | 0.663 |
|  | **29SP2EC_TGTT** | 38 | 38.000 | 19.000 | 3.360 | 0.316 | 0.947 | 0.960 | 0.667 |
| Population | Locus | N | Na | Ne | I | Ho | He | uHe | F |
|  | **SP2EC57_3mer** | 33 | 37.000 | 33.508 | 3.557 | 0.273 | 0.970 | 0.985 | 0.719 |
|  | **48SP2EC_ATTGA** | 40 | 32.000 | 9.816 | 2.956 | 0.200 | 0.898 | 0.909 | 0.777 |
|  | **SP2EC7_TCAAG** | 36 | 38.000 | 27.574 | 3.507 | 0.278 | 0.964 | 0.977 | 0.712 |
|  | **Ev2_TTGAA** | 14 | 11.000 | 7.396 | 2.224 | 0.071 | 0.865 | 0.897 | 0.917 |
|  | **SP2EC8_ATATG** | 30 | 37.000 | 33.962 | 3.563 | 0.233 | 0.971 | 0.987 | 0.760 |
|  | **Ev8_AATTG** | 26 | 25.000 | 23.719 | 3.195 | 0.038 | 0.958 | 0.977 | 0.960 |
| Porto, PXO, MAD | **SP2EC6_ATTGT** | 4 | 5.000 | 4.571 | 1.560 | 0.250 | 0.781 | 0.893 | 0.680 |
|  | **Ev1_TATGA** | 4 | 7.000 | 6.400 | 1.906 | 0.750 | 0.844 | 0.964 | 0.111 |
|  | **32SP2EC_TATGA** | 4 | 7.000 | 6.400 | 1.906 | 0.750 | 0.844 | 0.964 | 0.111 |
|  | **SP2EC9_ATATC** | 5 | 6.000 | 5.556 | 1.748 | 0.200 | 0.820 | 0.911 | 0.756 |
|  | **23SP2EC_ATGA** | 3 | 3.000 | 2.000 | 0.868 | 0.333 | 0.500 | 0.600 | 0.333 |
|  | **29SP2EC_TGTT** | 3 | 3.000 | 3.000 | 1.099 | 0.000 | 0.667 | 0.800 | 1.000 |
|  | **SP2EC57_3mer** | 3 | 3.000 | 3.000 | 1.099 | 0.000 | 0.667 | 0.800 | 1.000 |
|  | **48SP2EC_ATTGA** | 3 | 4.000 | 3.600 | 1.330 | 0.333 | 0.722 | 0.867 | 0.538 |
|  | **SP2EC7_TCAAG** | 3 | 5.000 | 4.500 | 1.561 | 0.667 | 0.778 | 0.933 | 0.143 |
|  | **Ev2_TTGAA** | 0 | 0.000 | 0.000 | 0.000 | 0.000 | 0.000 | 0.000 |  |
|  | **SP2EC8_ATATG** | 4 | 5.000 | 4.571 | 1.560 | 0.250 | 0.781 | 0.893 | 0.680 |
|  | **Ev8_AATTG** | 2 | 2.000 | 2.000 | 0.693 | 0.000 | 0.500 | 0.667 | 1.000 |
| Sesimbra, POR | **SP2EC6_ATTGT** | 10 | 4.000 | 2.439 | 1.018 | 0.400 | 0.590 | 0.621 | 0.322 |
|  | **Ev1_TATGA** | 0 | 0.000 | 0.000 | 0.000 | 0.000 | 0.000 | 0.000 |  |
|  | **32SP2EC_TATGA** | 0 | 0.000 | 0.000 | 0.000 | 0.000 | 0.000 | 0.000 |  |
|  | **SP2EC9_ATATC** | 7 | 3.000 | 2.333 | 0.956 | 0.000 | 0.571 | 0.615 | 1.000 |
|  | **23SP2EC_ATGA** | 7 | 3.000 | 1.782 | 0.759 | 0.286 | 0.439 | 0.473 | 0.349 |
|  | **29SP2EC_TGTT** | 9 | 7.000 | 4.909 | 1.754 | 0.222 | 0.796 | 0.843 | 0.721 |
|  | **SP2EC57_3mer** | 10 | 9.000 | 7.407 | 2.085 | 0.300 | 0.865 | 0.911 | 0.653 |
|  | **48SP2EC_ATTGA** | 10 | 7.000 | 3.846 | 1.634 | 0.500 | 0.740 | 0.779 | 0.324 |
|  | **SP2EC7_TCAAG** | 5 | 5.000 | 4.545 | 1.557 | 0.200 | 0.780 | 0.867 | 0.744 |
|  | **Ev2_TTGAA** | 0 | 0.000 | 0.000 | 0.000 | 0.000 | 0.000 | 0.000 |  |
|  | **SP2EC8_ATATG** | 0 | 0.000 | 0.000 | 0.000 | 0.000 | 0.000 | 0.000 |  |
|  | **Ev8_AATTG** | 4 | 3.000 | 2.667 | 1.040 | 0.000 | 0.625 | 0.714 | 1.000 |

**Table S3.** Estimate of null allele frequencies per population (first row) and locus (first column) sampled. Analysis based on 100 bootstraps. For abbreviations see Table 4.

|  | ***Calheta, PIX, AZO*** | ***Fonte, PIX, AZO*** | ***Furna, PIX, AZO*** | ***São Caetano, PIX, AZO*** | ***Morro da Enseada, SJZ, AZO*** | ***Dori, SMG, AZO*** | ***Ilhéu V. Franca, SMG, AZO*** | ***Lagoa, SMG, AZO*** | ***Porto, PXO, MAD*** | ***Ilhéu de Cima, PXO, MAD*** | ***Sesimbra, POR*** |
| --- | --- | --- | --- | --- | --- | --- | --- | --- | --- | --- | --- |
| **SP2EC52_3mer** | 0.06222 | 0.06502 | 0.10582 | 0.0023 | 0 | 0.02275 | 0.08483 | 0 | 0 | 0.06028 | 0.1194 |
| **SP2EC6_ATTGT** | 0.03026 | 0.06661 | 0.09155 | 0.07015 | 0.00001 | 0.08771 | 0.11832 | 0.00001 | 0.22265 | 0.15441 | 0.32514 |
| **32SP2EC_TATGA** | 0.19117 | 0.20472 | 0.08556 | 0.13215 | 0.1815 | 0.18274 | 0.17295 | 0.16222 | 0.00045 | 0.20336 | - |
| **Ev1_TATGA** | 0.19117 | 0.19516 | 0.08556 | 0.14594 | 0.1815 | 0.18274 | 0.17295 | 0.16222 | 0.00045 | 0.23433 | 0.16837 |
| **SP2EC9_ATATC** | 0.2792 | 0.2288 | 0.22765 | 0.20709 | 0.18649 | 0.18008 | 0.34623 | 0.26773 | 0.3659 | 0.32174 | - |
| **SP2EC57_3mer** | 0.29682 | 0.31476 | 0.39159 | 0.25124 | 0.25965 | 0.25662 | 0.15305 | 0.37121 | 0.3 | 0.31423 | - |
| **23SP2EC_ATGA** | 0.0689 | 0.10026 | 0.11783 | 0.08118 | 0.13422 | 0.17881 | 0.17146 | 0 | 0 | 0.27283 | 0.38687 |
| **48SP2EC_ATTGA** | 0.14207 | 0.20253 | 0.28409 | 0.16401 | 0.34161 | 0.18416 | 0.18742 | 0.19785 | 0 | 0.30693 | 0 |
| **29SP2EC_TGTT** | 0.21678 | 0.20517 | 0.35498 | 0.22747 | 0.31582 | 0.29803 | 0.2832 | 0.15216 | 0.4 | 0.30297 | 0.30314 |
| **SP2EC7_TCAAG** | 0.36836 | 0.27795 | 0.28829 | 0.31705 | 0.20094 | 0.3725 | 0.24537 | 0.17293 | 0.00058 | 0.32294 | 0.1 |
| **SP2EC8_ATATG** | 0.2773 | 0.40274 | 0.30658 | 0.40522 | 0.2521 | 0.28105 | 0.30566 | 0.26222 | 0.3 | 0.36409 | 0.3301 |
| **Ev2_TTGAA** | 0.00002 | 0.12578 | 0.001 | 0.09145 | 0.00001 | 0.10107 | 0.11254 | 0.15027 | - | 0.29037 | - |
| **Ev8_AATTG** | 0.32755 | 0.31999 | 0.36417 | 0.3925 | 0.30949 | 0.39824 | 0.45252 | 0.43223 | 0.22222 | 0.44226 | 0.36869 |

**Table S4.** Estimates of migration rates retrieved from multilocus genotype data of *Ervilia castanea* populations through BayesAss v3. Values reflect the proportion of individuals of the sink population that originate from the source. Populations in the first column indicate the source populations whilst population in the first row indicate the receiving population. Values and Standard Error (in brackets) reported. Values higher than 10% reported in bold.

|  | Calheta, PIX, AZO | Fonte, PIX, AZO | Furna, PXO, AZO | São Caetano, PIX, AZO | Morro da Enseada, SJZ, AZO | Dori, SMG, AZO | Ilhéu V. Franca, SMG, AZO | Lagoa, SMG, AZO | Ilhéu de Cima, PXO, MAD | Porto, PXO, MAD | Sesimbra, POR |
| --- | --- | --- | --- | --- | --- | --- | --- | --- | --- | --- | --- |
| Calheta, PIX, AZO | 0.6800 (0.0128) | 0.0574 (0.0255) | 0.0134 (0.0128) | 0.0401 (0.0213) | 0.0133 (0.0128) | 0.0533 (0.0240) | 0.0494 (0.0242) | 0.0133 (0.0128) | 0.0533 (0.0242) | 0.0133 (0.0128) | 0.0133 (0.0128) |
| Fonte, PIX, AZO | 0.0064 (0.0063) | 0.9249 (0.0193) | 0.0064 (0.0062) | 0.0064 (0.0062) | 0.0064 (0.0062) | 0.0170 (0.0106) | 0.0067 (0.0066) | 0.0064 (0.0063) | 0.0065 (0.0064) | 0.0064 (0.0063) | 0.0065 (0.0063) |
| Furna, PXO, AZO | 0.0152 (0.0145) | 0.1062 (0.0320) | 0.6818 (0.0145) | 0.0152 (0.0145) | 0.0151 (0.0144) | 0.0300 (0.0198) | 0.0151 (0.0144) | 0.0151 (0.0145) | 0.0761 (0.0290) | 0.0151 (0.0144) | 0.0151 (0.0145) |
| São Caetano, PIX, AZO | 0.0060 (0.0059) | 0.0059 (0.0058) | 0.0059 (0.0058) | 0.9405 (0.0169) | 0.0060 (0.0059) | 0.0059 (0.0058) | 0.0059 (0.0058) | 0.0059 (0.0058) | 0.0060 (0.0059) | 0.0060 (0.0058) | 0.0059 (0.0059) |
| Morro da Enseada, SJZ, AZO | 0.0152 (0.0146) | 0.0455 (0.0240) | 0.0152 (0.0145) | 0.0304 (0.0201) | 0.6818 (0.0145) | 0.0452 (0.0237) | 0.0457 (0.0241) | 0.0152 (0.0145) | 0.0755 (0.0291) | 0.0151 (0.0144) | 0.0151 (0.0145) |
| Dori, SMG, AZO | 0.0074 (0.0073) | 0.0146 (0.0106) | 0.0075 (0.0072) | 0.0120 (0.0100) | 0.0074 (0.0073) | 0.9102 (0.0221) | 0.0113 (0.0106) | 0.0074 (0.0072) | 0.0075 (0.0074) | 0.0073 (0.0072) | 0.0074 (0.0072) |
| Ilhéu V. Franca, SMG, AZO | 0.0076 (0.0074) | 0.0180 (0.0118) | 0.0076 (0.0074) | 0.0219 (0.0126) | 0.0075 (0.0073) | 0.0076 (0.0074) | 0.8996 (0.0233) | 0.0076 (0.0074) | 0.0076 (0.0074) | 0.0076 (0.0074) | 0.0075 (0.0074) |
| Lagoa, SMG, AZO | 0.0139 (0.0132) | 0.0140 (0.0135) | 0.0139 (0.0133) | 0.0966 (0.0301) | 0.0139 (0.0133) | 0.0139 (0.0134) | 0.0278 (0.0185) | 0.6805 (0.0133) | 0.0977 (0.0303) | 0.0139 (0.0133) | 0.0139 (0.0133) |
| Ilhéu de Cima, PXO, MAD | 0.0055 (0.0055) | 0.0056 (0.0055) | 0.0056 (0.0055) | 0.0056 (0.0055) | 0.0055 (0.0055) | 0.0056 (0.0055) | 0.0055 (0.0054) | 0.0056 (0.0055) | 0.9444 (0.0159) | 0.0056 (0.0055) | 0.0056 (0.0055) |
| Porto, PXO, MAD | 0.0207 (0.0195) | 0.0414 (0.0266) | 0.0208 (0.0196) | 0.0623 (0.0316) | 0.0208 (0.0195) | 0.0418 (0.0268) | 0.0208 (0.0195) | 0.0210 (0.0197) | 0.0420 (0.0270) | 0.6875 (0.0195) | 0.0209 (0.0196) |
| Sesimbra, POR | 0.0158 (0.0151) | 0.0213 (0.0189) | 0.0158 (0.0151) | 0.0317 (0.0209) | 0.0159 (0.0152) | **0.1112 (0.0336)** | 0.0158 (0.0151) | 0.0158 (0.0150) | 0.0581 (0.0277) | 0.0159 (0.0152) | 0.6826 (0.0152) |

**
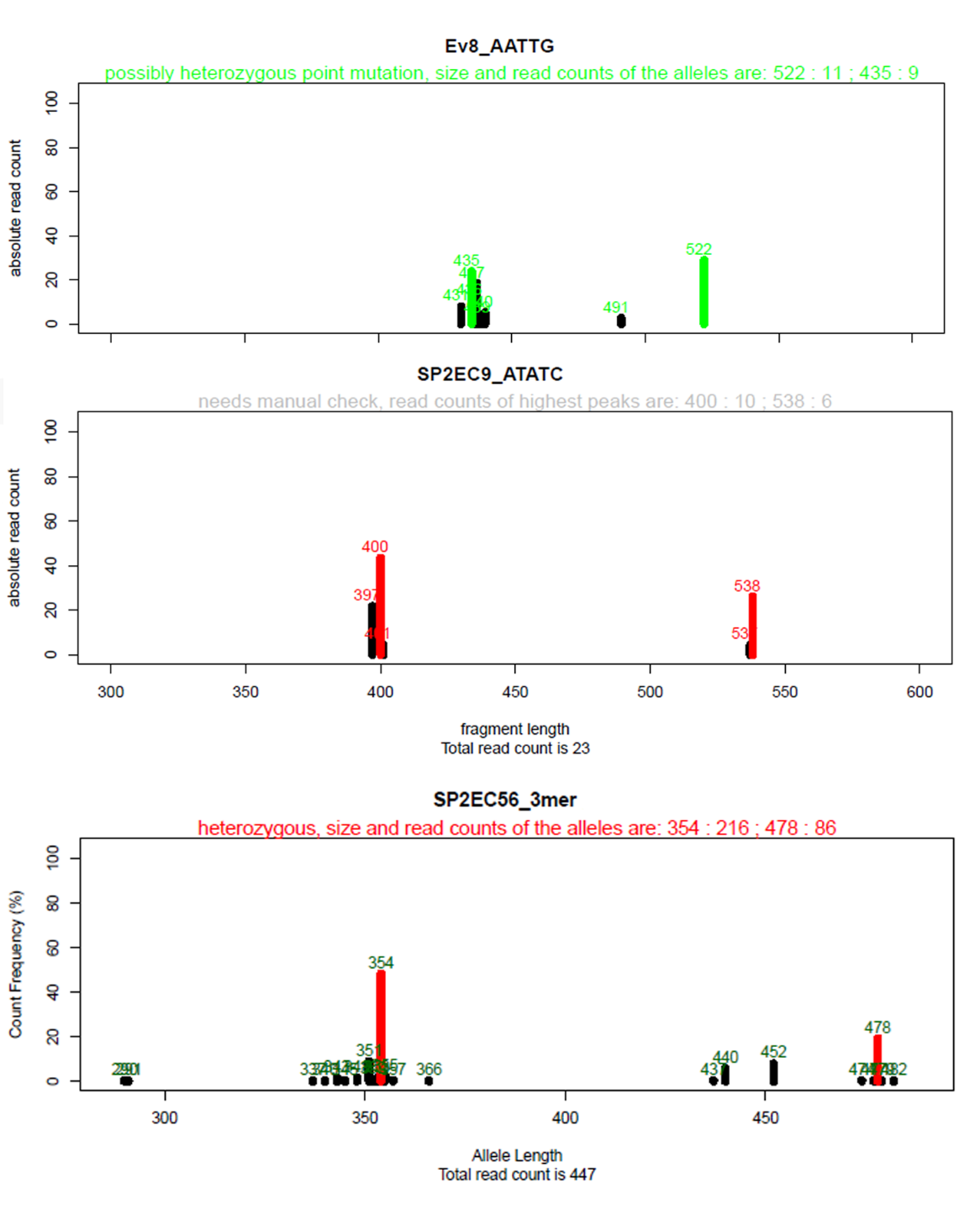
**

**Figure S1.** Examples of potential duplicated loci for markers EV8_AATG, SP2EC9_ATATC and SP2EC56_3mer within the WAI dataset of *Ervilia castanea*. Each plot shows at least two clearly distinct peaks of abundance read count or frequency count indicating co-amplification in the three analysed samples. Consistent co-amplification with more than one pattern with divergent lengths found within the dataset is hereby inferred as evidence of duplication.
